# Supplementary material for: Group antenatal care compared with standard antenatal care for Somali-Swedish women: a historically controlled evaluation of the Hooyo Project
Source: BMJ Open. 2023 Jan 25;13(1):e066000. doi: 10.1136/bmjopen-2022-066000 (PMC9884917; doi:10.1136/bmjopen-2022-066000)
Supplement: Author's manuscript [file bmjopen-2022-066000.draft_revisions.pdf]

BMJ Open

BMJ Open is committed to open peer review. As part of this commitment we make the peer review history of every article we publish publicly available.

When an article is published we post the peer reviewers' comments and the authors' responses online. We also post the versions of the paper that were used during peer review. These are the versions that the peer review comments apply to.

The versions of the paper that follow are the versions that were submitted during the peer review process. They are not the versions of record or the final published versions. They should not be cited or distributed as the published version of this manuscript.

BMJ Open is an open access journal and the full, final, typeset and author-corrected version of record of the manuscript is available on our site with no access controls, subscription charges or pay-per-view fees (http://bmjopen.bmj.com).

If you have any questions on BMJ Open's open peer review process please email info.bmjopen@bmj.com

Comparing group antenatal care and standard antenatal care for Somali-Swedish women. Findings from the Hooyo-project - a historically controlled evaluation.

Journal:	*BMJ Open*
Manuscript ID	bmjopen-2022-066000
Article Type:	Original research
Date Submitted by the Author:	09-Jul-2022

Complete List of Authors:	Ahrne, Malin ; Karolinska Institutet, Department of Women's and Children's Health<br>Byrskog, Ulrika; Dalarna University School of Education Health and Social Studies<br>Essen, Birgitta; Uppsala University, Women's and Children's Health<br>Andersson, Ewa; Karolinska Institutet, Department of Women's and Children's Health<br>Small, Rhonda; Karolinska Institutet, Department of Women's and Children's Health; La Trobe University<br>Schytt, Erica; Uppsala University Center for Clinical Research Dalarna; Western Norway University of Applied Sciences

Keywords:	Antenatal < GENETICS, Prenatal diagnosis < OBSTETRICS, Maternal medicine < OBSTETRICS, Reproductive medicine < GYNAECOLOGY, SOCIAL MEDICINE

SCHOLARONE™
Manuscripts

I, the Submitting Author has the right to grant and does grant on behalf of all authors of the Work (as defined in the below author licence), an exclusive licence and/or a non-exclusive licence for contributions from authors who are: i) UK Crown employees; ii) where BMJ has agreed a CC-BY licence shall apply, and/or iii) in accordance with the terms applicable for US Federal Government officers or employees acting as part of their official duties; on a worldwide, perpetual, irrevocable, royalty-free basis to BMJ Publishing Group Ltd ("BMJ") its licensees and where the relevant Journal is co-owned by BMJ to the co-owners of the Journal, to publish the Work in this journal and any other BMJ products and to exploit all rights, as set out in our licence.

The Submitting Author accepts and understands that any supply made under these terms is made by BMJ to the Submitting Author unless you are acting as an employee on behalf of your employer or a postgraduate student of an affiliated institution which is paying any applicable article publishing charge ("APC") for Open Access articles. Where the Submitting Author wishes to make the Work available on an Open Access basis (and intends to pay the relevant APC), the terms of reuse of such Open Access shall be governed by a Creative Commons licence – details of these licences and which Creative Commons licence will apply to this Work are set out in our licence referred to above.

Other than as permitted in any relevant BMJ Author's Self Archiving Policies, I confirm this Work has not been accepted for publication elsewhere, is not being considered for publication elsewhere and does not duplicate material already published. I confirm all authors consent to publication of this Work and authorise the granting of this licence.

For peer review only

Comparing group antenatal care and standard antenatal care for Somali-Swedish women.

Findings from the Hooyo-project - a historically controlled evaluation

Authors: Malin Ahrne[a], Ulrika Byrskog[b], Birgitta Essén[c], Ewa Andersson[a], Rhonda Small[ad], Erica Schytt[ef].

Affiliations:
[a]Department of Women's and Children's Health, Karolinska Institutet, Stockholm, Sweden
[b]School of Health and Welfare, Dalarna University, Falun, Sweden
[c]Women's and Children's Health, IMCH, Uppsala University, Sweden
[d]Judith Lumley Centre, La Trobe University, Melbourne, Australia
[e]Centre for Clinical Research Dalarna - Uppsala University, Falun, Sweden
[f]Faculty of Health and Social Sciences, Western Norway University of Applied Sciences, Norway

Contacts:

malin.ahrne@ki.se (M. Ahrne), uby@du.se (U. Byrskog), birgitta.essen@kbh.uu.se (B. Essén), Ewa.Andersson@ki.se (E. Andersson), R.Small@latrobe.edu.au (R. Small), Erica.Schytt@regiondalarna.se (E. Schytt)

Corresponding author:
Malin Ahrne
Department of Women´s and Children´s Health
Karolinska Institutet
Tomtebodavägen 18A
SE-171 77 Stockholm, Sweden
E-mail: malin.ahrne@ki.se

For peer review only

For peer review only - http://bmjopen.bmj.com/site/about/guidelines.xhtml

Licence Statement

I, the Submitting Author has the right to grant and does grant on behalf of all authors of the Work (as defined in the below author licence), an exclusive licence and/or a non-exclusive licence for contributions from authors who are: i) UK Crown employees; ii) where BMJ has agreed a CC-BY licence shall apply, and/or iii) in accordance with the terms applicable for US Federal Government officers or employees acting as part of their official duties; on a worldwide, perpetual, irrevocable, royalty-free basis to BMJ Publishing Group Ltd ("BMJ") its licensees and where the relevant Journal is co-owned by BMJ to the co-owners of the Journal, to publish the Work in BMJ Open and any other BMJ products and to exploit all rights, as set out in our licence.

The Submitting Author accepts and understands that any supply made under these terms is made by BMJ to the Submitting Author unless you are acting as an employee on behalf of your employer or a postgraduate student of an affiliated institution which is paying any applicable article publishing charge ("APC") for Open Access articles. Where the Submitting Author wishes to make the Work available on an Open Access basis (and intends to pay the relevant APC), the terms of reuse of such Open Access shall be governed by a Creative Commons licence – details of these licences and which Creative Commons licence will apply to this Work are set out in our licence referred to above.

For peer review only

Abstract

Objectives: Comparing language-supported group antenatal care (gANC) and standard antenatal care (sANC) for Somali-born women in Sweden, measuring overall ratings of care and emotional wellbeing, and testing the feasibility of the outcome measures.

Design: A historically controlled evaluation with one intervention and one control group, nested in an intervention development and feasibility study**.**

Setting: Midwifery-led antenatal care clinic in a mid-sized Swedish town.

Participants: Pregnant Somali-born women (<25 gestational weeks); 64 women in gANC and 81 in sANC.

Intervention: Language-supported gANC (2017-2019). Participants were offered seven 60-minute group-sessions with other Somali-born women led by 1-2 midwives, in addition to 15-30 minute individual appointments with their designated midwife.

Outcomes: Primary outcomes were women's overall ratings of antenatal care and emotional wellbeing (Edinburgh Postnatal Depression Scale (EPDS)) in gestational week ≥35 and 2-months postpartum. Secondary outcomes were specific care experiences, information received, social support, knowledge of pregnancy danger signs and obstetric outcomes.

Results: Recruitment and retention of participants was challenging. No relevant differences regarding overall ratings of antenatal care were detected. The reduction in mean EPDS-score was greater in the intervention group when adjusting for differences at baseline (mean difference -1.89; CI 95% -3.73 - -0.07). Women in gANC were happier with received pregnancy and birth information, e.g. caesarean section where 94.9% believed the information was sufficient compared to 17.5% in standard care ($p<0.001$) in late pregnancy.

Conclusions: This evaluation suggests some potential for language-supported gANC to improve knowledge acquisition among pregnant Somali-born women. Feasibility of assessing relevant care and obstetric outcomes with Somali-born women was demonstrated, but an adequately powered randomised trial is needed to evaluate the effectiveness of the intervention.

For peer review only - http://bmjopen.bmj.com/site/about/guidelines.xhtml

Strengths and limitations of this study

- This is the first evaluation of a group antenatal care intervention for migrant women in Sweden.
- This study was part of an intervention development and feasibility study powered to detect clinically relevant differences in women's overall ratings of antenatal care and the Edinburgh Postnatal Depression Scale (EPDS) but not its impact in relation to other outcomes.
- The study had a participatory approach that integrated the perspectives of midwives and Somali-born women in the intervention development and in the study design.
- Emotional wellbeing was measured with the EPDS, contributing to the evidence for its use as it is not yet validated for Somali-speaking women.

For peer review only

Introduction

To improve maternal health and to leave no one behind, there is a need for innovative evidence-based approaches to antenatal care (1-3). Migration interacts with other social health determinants contributing to health inequalities (4, 5). With a changing demography of pregnant women in many high-income countries, rethinking how care is provided to migrant women is necessary to improve pregnancy outcomes (3, 6, 7). Studies show that many migrant women in high-income countries are at an increased risk of poor pregnancy outcomes such as higher incidence of co-morbidities (8), pre-eclampsia (9), preterm birth (10-12), post-term birth (13, 14), lower use of some obstetrical interventions (15), higher risk of emergency caesarean birth, postpartum depression and maternal death (15), and for infants, higher risk of low Apgar score (11, 14), low birthweight (8, 11, 14, 16, 17), small for gestational age (SGA) (16, 17) and perinatal mortality (8, 10, 11, 18). [illegible]

For Somali-born women, higher rates of complications during pregnancy and birth compared to the general populations of migrant receiving countries have been reported, such as anaemia and severe hyperemesis (19), fetal distress, perineal laceration, postpartum hemorrhage (12), higher incidence of caesarean sections, SGA (19, 20) perinatal deaths (21) and stillbirths (22, 23). A lower rate of preterm birth in Somali-born women has been observed (16, 22, 24), and a higher incidence of post-term birth (20, 24). [illegible]

Qualitative studies (25-28) and systematic reviews show that migrant women more often report poor experiences of maternity care, such as communication problems (3, 29-31), lack of familiarity with care systems (3, 29, 30), sub-optimal care (3, 15, 30, 32) and discrimination (3, 15, 29, 31). The risk of maternal and reproductive health inequalities and sub-optimal care affecting Somali-born women has been described for decades (23, 33-38). Swedish studies show that migrant women (39), including Somali-born women (19), commence ANC later, make fewer ANC visits and are less likely to contact obstetric care for decreased fetal movements (20). Finally, lower attendance in childbirth preparation and parenting classes during pregnancy among migrant women in Sweden and elsewhere has also been reported (40-42).

New models of maternity care that address migrant women's specific socioeconomic and psychosocial circumstances have been proposed (3). One such model is group antenatal care (gANC), which typically incorporates pregnancy check-ups and group sessions for education

For peer review only - http://bmjopen.bmj.com/site/about/guidelines.xhtml

and social support in a group of women at similar stages of pregnancy (43, 44). Slightly different models of gANC have been developed in various settings (45-47). gANC has the potential to increase ANC attendance, improve satisfaction with care and pregnancy outcomes such as lower rates of preterm birth, increased breastfeeding rates and reduced risk of depressive symptoms (48-51), despite some inconsistency in the evidence (44, 47, 52). In a Swedish setting, low-risk women in gANC were more satisfied with the information received about labour and birth, and with the midwives´ engagement (53), however women with inadequate Swedish-proficiency were not included. Studies of gANC for migrant women are scarce, but there are some promising examples of small-scale interventions (7, 54, 55). None has been conducted in Sweden, where 26% of the population is foreign-born (56).

The aim of this study was to compare language-supported group antenatal care and standard antenatal care for Somali-born women in Sweden in terms of women's overall ratings of care and their emotional wellbeing in late pregnancy and 2-months postpartum, and to evaluate the feasibility of the outcome measures.

For peer review only

Methodology

Study design

A single-site historically controlled evaluation with one intervention and one control group was conducted between October 2016 and September 2019, as part of the Hooyo-project ('mother' in Somali), a development and feasibility study of gANC for Somali-born women in Sweden. The intervention group received gANC (May 2017 – September 2019) and the control group received standard antenatal care (sANC) (October 2016 – May 2017). Questionnaires were used at three time points to assess ratings of care, emotional wellbeing and a number of secondary outcomes. Additional data were retrieved from patient records. The study was registered in ClinicalTrials.gov (Identifier: NCT03879200).

Patient and public involvement

The intervention was developed in collaboration with midwives and members of the Somali community; the process is described in detail elsewhere (57). Initial focus group discussions with Somali women and men informed the intervention design (25). A study reference group included midwives and members of the Somali community with relevant professional backgrounds and was engaged in all stages of the intervention development. A bicultural research assistant was responsible for recruiting women to the study and conducting the interviews.

Participants and recruitment

Participants were pregnant (<25 gestational weeks (gwk)) Somali-born women. Exclusion criteria were severe health conditions (e.g. need of specialist obstetric care or a severe mental health condition). At the first ANC appointment, midwives provided initial oral and written information to all eligible women and if agreed, the bilingual research assistant then provided in-depth information, recruited those interested to the study and obtained consent. Information materials were available in Swedish and Somali.

Setting

Two ANC clinics were involved in developing the model and one clinic implemented the intervention. The implementing clinic is public and located in a mid-sized town in Sweden, with a mixed socioeconomic uptake area, and has 10 midwives.

For peer review only - http://bmjopen.bmj.com/site/about/guidelines.xhtml

Antenatal care in Sweden is free of charge with continuity of care throughout pregnancy, and referral to an obstetrician or other specialist when needed. A minimum of 8-9 midwife appointments is recommended (58), exclusive of ultrasound for pregnancy dating, which is recommended in gwk 18-20. Appointments are usually 30 minutes. A first, early visit focuses on lifestyle factors. The second visit is usually 45 minutes, including a detailed patient history. Visits 3-9 include health controls of the woman and unborn baby and provision of information, including information about danger signs like vaginal bleeding, leakage of amniotic fluids, decreased fetal movement and symptoms of preeclampsia and where to seek healthcare. Language interpretation can be arranged, either telephone or face to face. ANC clinics offer additional birth preparation in groups and parenting classes. Partners are welcome to attend both individual appointments and classes.

The Hooyo gANC-intervention

The intervention was a combination of group antenatal care and individual check-ups, with language support and integrated childbirth and parenting education. Participants were offered seven 60-minute group sessions together with other women of similar gestational age, led by 1-2 midwives. Group rules were set by participants, so partners were mostly but not always invited to participate. Directly before or after each group session, a 15-minute individual appointment was scheduled for health check-ups with the designated midwife. Participants started attending group antenatal care in gwk 20-26. Additional individual appointments were scheduled if needed. Frequency and total number of appointments followed the Swedish national ANC recommendations (58). The groups were fixed, meaning that women were assigned to a specific group with the same participants and a specific start and end date. Typically, the sessions started with a presentation of a selected topic, following Swedish national guidelines; life style, pregnancy, birth, practical birth preparations, the new-born baby, breast feeding (and alternatives), parenthood and relationships (58). The midwives were to be responsive to the interests and concerns of the group, and to encourage questions, dialogue etc. Language support was provided by a female interpreter in every group session, usually the same interpreter who was also a trained nurse assistant. The interpreter also served as a co-facilitator of the group. Before start-up, the midwives received 1 ½ - days training on person-centred care, use of motivational interviewing in groups and group dynamics. A manual was developed. The midwives planned the sessions and chose relevant pedagogical tools like films or anatomical models.

For peer review only - http://bmjopen.bmj.com/site/about/guidelines.xhtml

Control group

The control group received standard, midwifery-led individual care in accordance with Swedish national guidelines (58) as described above.

Data collection

Three questionnaires with both closed and open-ended questions were developed by the research team in English and translated to Swedish and Somali and are described in detail in the study protocol (57). Some questions from the Migrant Friendly Maternity Care Questionnaire (MFMCQ) were included but adapted slightly (59). The Edinburgh Postnatal Depression Scale (EPDS) was included in all three questionnaires (see below) (60).

A bilingual research assistant recorded women's responses to the questionnaires during face-to-face or telephone interviews; questionnaire one (Q1) in gwk 21-25, the second questionnaire (Q2) in late pregnancy (<gwk 35) and the third questionnaire (Q3) for the follow-up at two months postpartum.

Baseline information on women's health, such as age, obstetric history, height, weight, use of tobacco in early pregnancy, diabetes mellitus type 2 (ICD code O24.1), haemoglobin and S-ferritin were retrieved from patient records. Gestational age at first ANC visit is presented in intervals ≤12; 13-20 and 21-25 gwk (after which women were not eligible). Ultrasound for dating of pregnancy is recommended in Sweden in gwk 18-20 (58), making week 20 a reasonable upper limit for the second interval. The questionnaires included sociodemographic information (i.e., language proficiency, level of education, marital status, occupational and migration factors). Entire household monthly disposable income was self-reported in Swedish kronor (SEK). Household size is the self-reported number of persons living in the same household.

Primary outcomes

Women's overall rating of antenatal care was assessed in late pregnancy and 2-months postpartum with the core question 'When thinking about your overall experience of antenatal care — in general, have you been happy with the care that you have received?' with response alternatives *always, mostly* (happy with care) *and sometimes, rarely* and *never* (not happy with care).

For peer review only - http://bmjopen.bmj.com/site/about/guidelines.xhtml

The EPDS is a 10-item scale initially developed to screen for postnatal depression symptoms, which is validated for use in Swedish during pregnancy (61) and has been translated to Somali but not validated (62). The validated language versions of the EPDS are routinely used for screening of mothers postpartum in child health units in Sweden (63). The 10 items are scored 0-3 according to severity of the self-reported symptoms, with a maximum score of 30. The scale gives an indication of depressive symptoms over the last 7 days. In Sweden, ≥13 points has been validated as an optimal cut-off for detecting depression in pregnant women (61). The EPDS was used at baseline, in late pregnancy and postpartum.

Secondary outcomes

Women's ratings of different components of antenatal care in late pregnancy and 2-months postpartum were also assessed. A number of questions in this format were asked: *"Have you been happy with…?"* with response alternatives *always, mostly, sometimes, rarely* and *never*. Responses were dichotomised in two categories: *"always + mostly"* and "*sometimes + rarely + never"*). Women's ratings of receiving sufficient information about pregnancy, labour and birth as well as social support were assessed (response alternatives *yes* or *no)* in late pregnancy and two months postpartum. Social support during pregnancy was measured by modified questions from the Pregnancy Risk Assessment Monitoring System (PRAMS) (64).

Knowledge about danger signs (vaginal bleeding, leakage of amniotic fluids, decreased fetal movement, and severe headache) and where to seek healthcare with specific symptoms (General Practitioner, labour ward, emergency room etc.) were assessed in Q2, in the question format *"What would you do if you experienced…."* with the following response alternatives *wait/self-care; contact the ANC midwife; general practitioner (GP) or nurse; delivery ward or emergency ward*.

Obstetric outcomes included: antenatal care parameters (number of ANC visits, number of visits to specialist care, referral to an obstetrician, asked about experience of violence, attendance at parent education), health parameters (Haemoglobin (lowest value and last value prior to birth), S-Ferritin (lowest value), weight gain during pregnancy, gestational diabetes mellitus (ICD-10 code O24.4)), birth outcomes (induction of labour, oxytocin for dystocia, pain relief, mode of birth, perineal injury, blood loss, breast feeding at the labour ward, length of stay), attendance at postpartum check-up, breastfeeding and BMI at the postpartum visit.

For peer review only - http://bmjopen.bmj.com/site/about/guidelines.xhtml

The number of ANC visits is presented as the median number of visits, and the proportion of women having 6 or fewer visits, 7-11 visits, or more than 11 visits.

Infant outcomes: gestational age, birthweight, SGA (ICD-10 codes P05.0 and P05.1), large for gestational age (LGA) (ICD-10 codes P08.0 and P08.1), Apgar score <7 at five minutes, umbilical cord pH (arterial and venous) and neonatal intensive care.

Sample size calculation

The study was designed to have power to detect clinically relevant differences in women's overall ratings of antenatal care and a difference in means on the EPDS. The initial sample size calculation of 70 women in each group (with 80% power and an alpha of 20%) was based on a national population study on Swedish speaking women's satisfaction with ANC two months postpartum (65). Our assumption was that the ratings of care would improve, from 65% of women receiving individual care being happy with the ANC care received (*"always + mostly happy with care"*) to 82% of those receiving gANC.

To have similar power to detect differences in EPDS mean scores, 63 women were required in each group, based on a hypothesized reduction from a mean of 8.0 in the control group to that of 6.0 in the intervention group (66). To allow for loss to follow-up with 20%, a total of 174 women needed to be recruited.

Statistical analyses

Women who were recruited to the intervention and control groups and contributed data were analysed according to the intention-to-treat (ITT) concept (67). Frequencies and percentages are reported for dichotomous variables, median and interquartile ranges (IQR) are reported for continuous variables. Chi-square tests were performed to test hypotheses for dichotomous variables, Mann-Whitney U tests for continuous variables. For the primary outcome "overall ratings of care" odds ratios were calculated.

An ANCOVA test was performed to test the difference in mean EPDS scores between gANC and standard care at two months postpartum, adjusted for differences in EPDS at baseline, after the exclusion of women with missing EPDS values (gANC n=41, sANC n=38). P-values <0.05 were deemed statistically significant and all tests were two tailed. All statistical analyses were performed in R version 4.01.

For peer review only - http://bmjopen.bmj.com/site/about/guidelines.xhtml

Results

Of 270 eligible women, 145 women were recruited to the study (53.7%) – 64 women to gANC and 81 to sANC. The number of women who declined to participate was 106 (39.3% of all eligible women), and 19 women did not meet the inclusion criteria (n=6) or were excluded for other reasons (n=13), such as moving or having a miscarriage.

The first questionnaire (Q1) was completed by 129 women (89% of all women recruited to the study) (gANC n=62; sANC n=67); the second questionnaire (Q2) by 80 women (55%) (gANC n=40; sANC n=40) and the third (Q3) by 86 women (59%) (gANC n=44; sANC n=42). Of the women in gANC and sANC, 38 women (59.4%) and 32 women (39.5%) responded to all three questionnaires respectively.

Baseline characteristics for the sample are described in Table 1. The groups were largely similar, however, a larger proportion of women in gANC had less than six years of education, spoke Somali well or fluently, had no previous births in Sweden and were involved in home duties, on parental leave or were unemployed. The median length of residence in Sweden in this sample was seven years.

Table 1 Characteristics of the sample

	Group antenatal care (n=63)		Standard antenatal care (n=73)	
	n (%)	Median; (IQR)	n (%)	Median; (IQR)
**Sociodemographics**				
Age (years)		31 (26.3; 33.8)		30 (25.0; 33.3)
Language proficiency, well/fluent				
Swedish	41 (65.1)		41 (56.2)	
Somali	62 (98.4)		60 (82.2)	
English	15 (23.8)		14 (19.2)	
Missing	1 (1.6)		8 (11.0)	
Completed level of education				
<6 years	32 (50.8)		25 (34.2)	
7-9 years	14 (22.2)		18 (24.7)	
>10 years	14 (22.2)		13 (17.8)	
Missing	3 (4.8)		17 (23.3)	
Marital status				
Married/engaged/in a relationship	58 (92.1)		66 (90.4)	
Divorced/widowed/single	4 (6.3)		0 (0.0)	
Missing	1 (1.6)		7 (9.6)	
Living with husband or partner	49 (78.8)		55 (75.3)	
Missing	14 (22.2)		18 (24.7)	
Household size		3 (2.0; 5.0)		4 (3.0; 6.0)

For peer review only - http://bmjopen.bmj.com/site/about/guidelines.xhtml

Occupation				
Employed	12 (19.0)		12 (16.4)	
Student	19 (30.2)		27 (37.0)	
Home duties/parental leave/unemployed	30 (47.6)		23 (31.5)	
Missing	2 (3.2)		11 (15.1)	
Entire household monthly disposable income (SEK)		19,000 (13,000; 31,750)		22,000 (19,000; 28,000)
Migration				
Length of residence (years)		7 (5.0; 8.8)		7 (4.0; 8.0)
Reason for migration				
Refugee/asylum seeker	32 (50.8)		30 (41.1)	
Family ties	30 (47.6)		34 (46.6)	
Other	0 (0.0)		1 (1.4)	
Missing	1 (1.6)		8 (11.0)	
Current migration status				
Asylum seeker	4 (4)		1 (1.4)	
Permanent residency	35 (55.6)		42 (57.5)	
Swedish citizen	23 (36.5)		22 (30.1)	
Missing	1 (1.6)		8 (11.0)	
**Obstetric history**				
Parity				
0	11 (17.5)		14 (19.2)	
1-2	18 (28.6)		18 (24.7)	
3-4	18 (28.6)		17 (23.3)	
≥5	15 (23.8)		18 (24.7)	
Missing	1 (1.6)		6 (8.1)	
Previous stillbirths/neonatal deaths	4 (6.5)		4 (5.8)	
No previous birth in Sweden	5 (7.9)		0 (0.0)	
**Current pregnancy**				
Gestational age (weeks) at first ANC visit		11 (8.8; 14.0)		12 (10.0; 14.0)
≤12	32 (51.6)		38 (55.1)	
13-20	14 (22.6)		21 (30.4)	
≥ 21 - 25 (after that not eligible)	2 (3.2)		1 (1.4)	
Missing	14 (22.6)		9 (13.0)	
Body Mass Index (kg/m²) at first visit		27.8 (24.3; 31.4)		27.6 (24.7; 31.3)
Underweight (<18.5)	3 (4.8)		4 (5.8)	
Normal weight (18.5-24.9)	14 (22.6)		16 (23.2)	
Overweight (25.0-29.9)	17 (27.4)		23 (33.3)	
Obesity (≥30)	20 (32.3)		22 (31.9)	
Missing	8 (12.9)		4 (5.8)	
Tobacco use in early pregnancy	0		0	
S-Ferritin at first visit		23.5 (15.8; 54.0)		23.5 (13.8; 42.8)
S-Haemoglobin at first visit		121.0 (113.3; 124.8)		121.5 (117.3; 126.8)
Diabetes mellitus type 2 prior to pregnancy	1 (1.6)		1 (1.4)	

For peer review only

Overall ratings of care

Women's overall rating of antenatal care was assessed through the core question *'When thinking about your overall experience of antenatal care in general, have you been happy with the care that you have received?'* in late pregnancy and 2-months postpartum, with the response alternatives *always, mostly*, *sometimes, rarely* and *never*. The vast majority of women in both groups responded *always* and some responded *mostly.* Very few women (5%), and only in the control group, responded *sometimes*, and there were no responses in the categories *rarely* and *never* (Figure 1). When the variable was dichotomised following the protocol, all women (100%) in the gANC group were happy (*always* + *mostly*) with the care they had received when asked in late pregnancy, compared to 96% in the control group (sANC). Similarly, two months postpartum, all (100%) of women who participated in gANC were happy with the care they had received, compared to 95% of women in sANC (statistical test not applicable). There were no statistical differences between the groups at either time point when the outcome measure was dichotomised into *always* versus *all other alternatives* (late pregnancy OR 1.42, CI 95% 0.50-4.16 and 6-8 weeks postpartum OR 2.71, CI 95% 0.88-9.41).

Figure 1. Women´s overall ratings of ANC care – the proportion of women who were *"always"*, *"mostly"*, *"sometimes"*, *"rarely"* and *"never"* happy with the care they received in the two different care models, assessed in late pregnancy and two months postpartum.

For peer review only

For peer review only - http://bmjopen.bmj.com/site/about/guidelines.xhtml

Emotional wellbeing

At baseline, 61 (98.4%) women in gANC and 64 (95.5%) women in sANC responded to all ten EPDS questions. In late pregnancy, 39 (97.5%) women in gANC and 37 (92.5%) women in sANC responded, and at the last measurement 2-months postpartum, 41 women in both groups respectively responded to all items (gANC 93.2%; sANC 97.6%).

Mean EPDS scores were higher in women in gANC than in women in sANC at baseline (gANC 9.19; sANC 5.94; diff in means 3.26; CI 95% 1.56-4.96) and in late pregnancy (gANC 8.51; sANC 5.73; diff means 2.78; CI 95% 0.62-4.95). Two months postpartum, the mean EPDS score was similar between the groups (gANC 3.90; sANC 5.00; diff means -1.1; CI 95% -2.80-0.61). An ANCOVA test of differences in the reduction of mean values (adjusted for differences at baseline) was made for women who responded to all three questionnaires (gANC n=41; sANC n=38). The reduction in mean EPDS-score was greater in the intervention group when adjusting for differences at baseline (mean difference -1.89; CI 95% -3.73 – -0.07).

Figure 2. Median scores on the Edinburgh Postnatal Depression Scale (EPDS); at baseline (Q1), late pregnancy (Q2) and 2 months postpartum (Q3) analysed by ANCOVA test of differences. The boxplot also shows minimum and maximum score, and the 1st and 3rd quartile and the mean differences with 95% CI at each time point.

We also checked the mean EPDS score of women who only responded to Q1. Their mean EPDS score was 7.2 compared to 7.5 among of all women included in Figure 2.

Secondary outcomes

Ratings of care

Women's ratings of specific components of antenatal care in relation to care model in late pregnancy and two months postpartum are presented in Table 2. Few statistically significant differences could be detected between the groups. Women in gANC were slightly happier with the advice they had received about how to manage common pregnancy disorders and they were more likely to report that the midwives had been encouraging and supportive (Table 2). No woman reported experience of negative discrimination by the midwives based on ethnicity, religion, culture, language etc.

For peer review only - http://bmjopen.bmj.com/site/about/guidelines.xhtml

Women's ratings of care during labour and birth as well as during the postpartum stay were also assessed at two months postpartum, and no differences between the groups were detected. Higher proportions of women in gANC, were *always or mostly happy with care* compared with women in sANC, both during labour and birth (gANC 60.3% (n=38); sANC 49.3% (n=36); p=0.423) and on the postpartum ward (gANC 63.5% (n=40); sANC 50.7% (n=37); p=0.218) though neither difference was statistically significant (not presented in table).

Table 2. Women's ratings of satisfaction with specific components of antenatal care (*"always or mostly happy with care"*) in relation to care model in late pregnancy and two months postpartum.

	Late pregnancy			Two months postpartum		
	Group antenatal care n (%)	Standard antenatal care n (%)	p-value	Group antenatal care n (%)	Standard antenatal care n (%)	p-value
Specifically, were you happy with…						
how the health of the baby was monitored during pregnancy?	40 (100.0)	38 (95.0)	0.990	44 (100.0)	42 (100.0)	NA
how your own health was monitored during pregnancy?	40 (100.0)	37 (94.9)	0.463	44 (100.0)	40 (95.2)	0.454
the management of your physical health?[1]	40 (100.0)	35 (89.7)	0.117			
the support you received to care for your own health? (i.e. life style factors)	39 (97.5)	32 (82.1)	0.057	42 (95.5)	38 (90.5)	0.629
the advice you received to manage common pregnancy disorders?	37 (92.5)	29 (74.4)	0.061	44 (100.0)	34 (81.0)	0.008
Did the ANC midwives…						
listen to your own concerns?	40 (100.0)	38 (100.0)	NA	44 (100.0)	40 (95.2)	0.454
make your partner feel welcomed and included?	37 (92.5)	34 (97.1)	1.000	40 (93.0)	39 (92.9)	1.000
show respect?[1]	39 (97.5)	37 (94.9)	0.982			
make you feel welcomed?[1]	39 (97.5)	39 (100.0)	1.000			
make you feel negatively discriminated against (because of ethnicity, religion, culture, language, etc.)?[1]	0 (0.0)	0 (0.0)				
have enough time for you during your visits?[1]	40 (100.0)	39 (100.0)	NA			
take your worries seriously?[1]	40 (100.0)	34 (91.9)	0.212			
keep you informed about the progress of your pregnancy?[1]	40 (100.0)	38 (97.4)	0.990			
been encouraging and supportive?[1]	40 (100.0)	32 (82.1)	0.016			
Did you feel comfortable to ask questions?[1]	40 (100.0)	39 (100.0)	NA			
Did you understand the information that you received from the midwives?[1]	40 (100.0)	38 (100.0)	NA			
Do you think that that the information you received from the midwives was relevant?[1]	39 (100.0)	37 (92.5)	0.248			

[1] Only in questionnaire 1

Sufficient information and social support

Both in late pregnancy and two months postpartum, women in gANC were generally happier with the information received on different aspects of pregnancy, labour and birth, breastfeeding, the care of the newborn baby and physical and emotional changes. Far more

For peer review only - http://bmjopen.bmj.com/site/about/guidelines.xhtml

women in gANC were happy with information about caesarean section (gANC 94.9%; sANC 17.5%; p<0.001), on the management of FGM/C (gANC 79.5%; sANC 40.0%; p=0.001) and the father's/partner's role during labour and birth (gANC 94.6%; sANC 42.5%; p<0.001) (Table 3) than women in sANC.

Table 3 also shows how women viewed the social support they received. No differences between the groups were observed when assessed in late pregnancy. At two months postpartum, 97.6% of women who had attended gANC reported that they had someone who could help with a temporary place to live if they should need it, compared to 25.0% of women in standard ANC (p<0.001). Additionally, 95.2% in gANC reported that they had made new friends through the ANC clinic during pregnancy, compared to 43.9% of women in standard care (p<0.001).

For peer review only

Table 3. Sufficient information and social support comparing gANC with sANC care

	Late pregnancy			Two months postpartum		
	Group antenatal care n (%)	Standard antenatal care n (%)	p-value	Group antenatal care n (%)	Standard antenatal care n (%)	p-value
**Sufficient information**						
Ratings for having received enough information about (yes vs no)						
why it is important to take iron tablets	39 (100.0)	38 (95.0)	0.368	43 (97.7)	39 (92.9)	0.576
when to go to hospital for labour	39 (100.0)	27 (67.5)	<0.001	44 (100.0)	37 (90.2)	0.107
management of FGM/C	31 (79.5)	16 (40.0)	0.001	39 (88.6)	21 (51.2)	<0.001
management of a delivery	36 (94.7)	17 (42.5)	<0.001	44 (100.0)	27 (64.3)	<0.001
pain relief during labour	38 (97.4)	12 (30.0)	<0.001	44 (100.0)	29 (69.0)	<0.001
induction of labour	33 (84.6)	11 (27.5)	<0.001	42 (95.5)	25 (59.5)	<0.001
vacuum extraction	33 (84.6)	6 (15.0)	<0.001	41 (93.2)	19 (45.2)	<0.001
caesarean section	37 (94.9)	7 (17.5)	<0.001	43 (97.7)	22 (52.4)	<0.001
father's/partner's role during labour	35 (94.6)	17 (42.5)	<0.001	43 (97.7)	26 (61.9)	<0.001
care at the neonatal care unit	38 (97.4)	21 (52.5)	<0.001	43 (97.7)	31 (73.8)	0.004
breastfeeding	38 (97.4)	23 (57.5)	<0.001	44 (100.0)	33 (78.6)	0.004
care of the newborn baby	38 (60.3)	21 (28.8)	<0.001	42 (97.7)	34 (81.0)	0.031
care for you after labour/who to contact after discharge	37 (94.9)	15 (37.5)	<0.001	44 (100.0)	29 (72.5)	0.001
physical changes during pregnancy[1]	39 (100.0)	30 (75.0)	0.004			
emotional changes during pregnancy[1]	37 (94.9)	29 (72.5)	0.001			
taking care of your own health during pregnancy?[1]	39 (100.0)	35 (87.5)	0.074			
when to contact the midwife or hospital between the scheduled ANC visits[1]	39 (100.0)	32 (80.0)	0.013			
physical changes after pregnancy[2]				44 (100.0)	29 (69.0)	<0.001
emotional changes after pregnancy[2]				42 (95.5)	24 (57.1)	<0.001
taking care of your own health after pregnancy[2]				43 (97.7)	28 (66.7)	<0.001
Do (did) you feel well prepared for labour and birth?	39 (100.0)	36 (90.0)	0.128	41 (97.6)	34 (85.0)	0.099
**Social support**						
Do you have someone						
to socialise with	38 (97.4)	36 (100.0)	1.000	42 (100.0)	41 (100.0)	NA
to talk with about your problems	38 (97.4)	37 (100.0)	1.000	42 (100.0)	41 (100.0)	NA
to help you if you were sick and needed to be in bed	38 (97.4)	37 (100.0)	1.000	42 (100.0)	41 (100.0)	NA
who can lend you 500 SEK if you have a sudden need?	38 (97.4)	35 (94.6)	0.963	42 (100.0)	41 (100.0)	NA
who can take care of your children for a while if needed?	37 (97.4)	29 (96.7)	1.000	42 (100.0)	38 (97.4)	0.970
who can help you with a temporary place to live next year if you should need it	31 (79.5)	28 (75.7)	0.902	40 (95.2)	18 (43.9)	<0.001
Have you made new friends through the antenatal care clinic during your pregnancy?[2]				41 (97.6)	10 (25.0)	<0.001
Have you felt lonely and isolated since you had the baby?[2]				1 (2.4)	2 (4.9)	0.983

[1]Only in questionnaire 1

[2]Only in questionnaire 2

For peer review only - http://bmjopen.bmj.com/site/about/guidelines.xhtml

Knowledge of danger signs

Knowledge of danger signs and who to contact if experiencing severe symptoms, was assessed in late pregnancy (not presented in table). A significantly higher proportion of women in gANC responded that they would contact the delivery ward in case of vaginal bleeding (gANC 54.0%; sANC 28.8%; p=0.004) or leakage of amniotic fluid (gANC 55.6%; sANC 26.0%; p=0.003). For severe headache and changes in vision, 46.0% of women in gANC said they would contact the emergency room compared to 23.3% of women in standard care (p=0.037). Of women in gANC, 30.2% responded that they would contact the delivery ward if experiencing reduced fetal movements, compared to 17.8% of women in sANC (p=0.526).

Obstetric outcomes

Obstetric outcomes in relation to care model are described in Table 4. Compared with women in standard care, women in gANC made more visits to the antenatal clinic and to specialist care, however, the other health parameters were similar between the groups. Labour and birth outcomes were also similar between the groups, such as induction of labour, pain relief, perineal injury, blood loss and length of stay.

For peer review only

Table 4. Obstetric outcomes in relation to care model

	Group antenatal care n=63		Standard care n=73		
	n (%)	median, IQR	n (%)	median, IQR	p-value
**Antenatal care**					
Number of ANC visits[1,2] (median, IQR)		9.5 (8.00; 11.00)		9.0 (7.25; 10.00)	0.022
≤6	3 (5.6)		2 (3.0)		0.018
7-11	40 (74.1)		61 (92.4)		
>11	11 (20.4)		3 (4.5)		
Number of visits to specialist care (median, IQR)		2.00 (1.00; 5.00)		1.00 (0.00; 3.00)	0.014
Number of women referred to a specialist (obstetrician)	49 (90.7)		51 (77.3)		0.085
Screened for experience of violence[3]	37 (88.1)		37 (84.1)		0.822
Attendance at parent education[4]	9 (27.3)		3 (8.3)		0.056
**Health parameters**					
Haemoglobin (lowest value)		111 (106; 118)		108 (104; 114)	0.181
Haemoglobin (last value prior to birth)		122 (118; 128)		120 (115; 126)	0.218
S-Ferritin (lowest value)		24 (16; 54)		23 (13; 42)	0.539
Weight gain during pregnancy (kg)		8.0 (4.3; 12.0)		7.0 (4.0; 12.0)	0.589
Gestational diabetes mellitus	7 (11.1)		7 (9.6)		0.771
**Labour and birth**					
Induction of labour	8 (17.8)		10 (16.4)		1.000
Oxytocin for dystocia	16 (42.1)		23 (37.7)		0.823
Pain relief					
Epidural (EDA)	9 (14.5)		5 (7.4)		0.259
Gas (Nitrous oxide)	33 (53.2)		35 (50.7)		0.912
Other (TENS, sterile water injection, bath, PCB, PDB)	1 (1.6)		2 (2.9)		1.000
Mode of birth					
Vaginal	42 (77.8)		52 (78.8)		0.064
Assisted vaginal delivery	0 (0.0)		6 (9.1)		
Elective caesarean	8 (14.8)		4 (6.1)		
Emergency caesarean	4 (7.4)		4 (6.1)		
Perineal injury (degree III-IV)	0 (0.0)		4 (5.8)		0.121
Blood loss (within 2 hours) (ml)		480 (300; 650)		400 (300; 538)	0.345
Breastfeeding on delivery ward	50 (98.0)		61 (96.8)		1.000
Length of stay (in hospital after birth)		1.00 (0.0; 2.0)		1.00 (0.0; 2.3)	0.590
Discharged the same day as giving birth	23 (43.4)		30 (46.9)		0.850
**Infant outcomes**					
Live births[5]	62		73		
Stillborn	1 (1.6)		1 (1.4)		1.000
Gestational age (weeks)		40.0 (39.0; 40.0)		40.0 (39.0; 40.3)	0.989
Birthweight (grams)		3580 (3180; 3778)		3490 (3166; 3776)	0.839
Small for gestational age	7 (11.1)		0 (0.0)		0.011
Large for gestational age	3 (4.7)		2 (2.7)		0.532
Apgar score <7 at 5 minutes	0 (0.0)		1 (1.7)		1.000
Umbilical cord pH (arterial)(median, IQR)		7.24 (7.20; 7.31)		7.26 (7.23; 7.31)	0.224
Umbilical cord pH (vein)(median, IQR)		7.35 (7.29; 7.37)		7.35 (7.30; 7.37)	0.795
Neonatal intensive care	1 (1.6)		5 (7.2)		0.212
**Postnatal check-up**					
Postnatal visit	51 (81.0)		47 (64.4)		0.032
Fully breastfeeding at 4 weeks after birth	25 (39.7)		24 (32.9)		0.410
Body Mass Index (kg/m2) at postnatal check-up visit		28.34 (25.56;31.64)		29.63 (26.44; 33.12)	0.379

[1]Not adjusted for gestational age.

[2]The recommended number of prenatal visits in Sweden is 9 for a normal 40-week pregnancy

For peer review only

For peer review only - http://bmjopen.bmj.com/site/about/guidelines.xhtml

[3]Screening for violence is recommended in the Swedish National Guidelines for ANC

[4]Other than group antenatal care

[5]One twin birth in standard care

Discussion

The aim of this study was to evaluate a model of gANC for Somali-speaking women in Sweden. No differences regarding overall ratings of antenatal care were detected, however, the reduction in mean EPDS-score was greater in the intervention group when adjusting for differences at baseline. In late pregnancy, women in gANC were happier with received pregnancy and birth information compared to women in standard care.

Participants in gANC rated their antenatal care experience at least on par with women who received standard antenatal care. The decrease in EPDS mean scores was larger in women in gANC than in sANC, adjusted for differences at baseline. The gANC group had relatively high EPDS median scores (10), when entering the intervention, and higher than the control group (6), indicating a probable selection bias. It is possible that women who were feeling vulnerable and wanting more support and information from ANC were more interested in participation in gANC. Women who participated in gANC had comparatively low levels of education, no previous births in Sweden, were less involved in working life and perhaps less knowledgeable about Swedish health care. Whether or not the larger reduction in mean EPDS scores among women in gANC can be attributed to the intervention or to other aspects of care given the study design, the reduced sample-size and the above discussion, is uncertain. In a small Norwegian study on postpartum depression among Somali women, the mean EPDS score was 2.97 (SD 3.31), which is very low, and might reflect a different understanding of the EPDS questions (68) or a selection of women other than in the present study. In our case, the EPDS questions were asked by the research assistant rather than women completing the EPDS themselves which may have impacted the responses, but it did allow for clarification of items, if they were not clear. Information on history of mental illness or medication was not collected. A still unpublished Swedish study suggests that the Somali version of the EPDS has some major lexical problems. During think-aloud interviews, some Somali-speaking women left items unanswered when responding to the EPDS because they did not understand the meaning of them (oral communication Schytt el al), which may also explain some of the extensive missing rates in other surveys using the scale. Challenges with interpretation in research on mental ill health and cultural aspects of mental health also needs to be considered (69). The EPDS has been translated into Somali but is not yet validated (62), so in the absence

For peer review only - http://bmjopen.bmj.com/site/about/guidelines.xhtml

of validation, caution is required when interpreting EPDS for this group. Additional or other outcome measures than the EPDS might be appropriate in future trials to evaluate the effects of gANC on Somali women's emotional wellbeing.

The most interesting differences detected between the gANC and sANC groups were related to secondary outcomes. Women attending gANC were more likely to say that they had received sufficient information than women in sANC on most aspects of childbirth studied. Findings on the women's knowledge of danger signs were also promising. A higher proportion of women in gANC gained a better understanding of which health care facility to turn to for danger signs like vaginal bleeding and leakage of amniotic fluid. Information and education have been identified as essential in midwifery-led antenatal care, for adequate knowledge and for women to better understand the health care system, and women prefer health professionals who combine clinical knowledge and skills with interpersonal and cultural competence (70). In this study, as in others, Somali-born women had a high prevalence of risk factors during pregnancy, for example previous perinatal mortality and obesity. Our findings suggest some potential for gANC to improve information provision and knowledge acquisition among pregnant Somali-born women, especially women resident in Sweden <10 years.

Additionally, gANC may have played a role in strengthening social networks as intended. Nearly all women in gANC said they had someone who could help with a temporary place to live should they need it, compared to 25.0% of women in standard care two months postpartum. Additionally, nearly all women in gANC reported that they had made new friends through the ANC clinic during pregnancy, compared to only half of women in standard care.

The women in gANC had more appointments with the midwife compared to women in standard care, possibly because individual appointments were shorter in gANC. As a result, additional appointments were booked, rather than extending the time for each appointment when more time was needed. gANC is a complex intervention (71) and it is therefore not possible, nor appropriate, to determine the separate contributions of different components. The components have been studied together because of their hypothesised capacity jointly to make a difference to the provision of care. A case study from the US suggests that an underlying mechanism in which group prenatal care is effective is through increased quantity and quality of patient and practitioner time together and improved communication, fostering

For peer review only - http://bmjopen.bmj.com/site/about/guidelines.xhtml

greater opportunity for cross-cultural exposure and decreasing factors like the clinician´s implicit or explicit bias and racism (72). A process evaluation of the Hooyo-project has assessed the overall feasibility of developing, implementing and testing gANC for Somali-Swedish women, including the perspectives of the midwives, where the different components of this gANC model have been examined in more depth (To be published Ahrne et al).

The inconsistent evidence about the impact of gANC on improved ratings of care and obstetric outcomes, may also be due to substantial differences between settings in the quality of standard antenatal care, as suggested in a Canadian study that found that group antenatal care was comparable to individual care on most outcomes measured (47). In the US however, there are greater disparities in the care received between subgroups, such as for women from minority groups or with low socioeconomic status who are uninsured (73).

To achieve greater prenatal health equity for migrant women, there is a need to continue to investigate specific interventions in antenatal care focused on pregnant women and their families, on maternity care health professionals and on the system in which they work.

Strengths and limitations

This is the first controlled evaluation of gANC in Sweden attempting to address particular needs of migrant women, with a participatory approach. This is of particular importance in groups of women who are often excluded in research, are hard to reach or when studying sensitive health issues (74). Moreover, this intervention and the evaluation were conducted in a real-world setting. Recruitment and retention of participants proved challenging; the response rate for the second questionnaire was 55% and 59% for the third questionnaire. The two-stage recruitment process with midwives providing information first and then recruitment of those showing initial interest subsequently by a bilingual research assistant may have made it harder to recruit women. Direct contact first with a Somali speaking research assistant may have made it simpler for women to receive enough information in their own language to agree to participate. We did not reach the recruitment goal of 70 women in each group, reducing the power to detect differences on one of our primary outcomes of overall rating of care between the groups. For the EPDS, however, we reached our recruitment goal of 63 women in each group. While the research assistants were pivotal in recruitment, retention and data collection in this study, women's responses were not given anonymously, which may have affected participation and responses. Additionally, participation in research was likely to be unfamiliar

For peer review only

for some of the women. These and other methodological challenges encountered in the study will be explored and reported separately. For this study, women were recruited from one site and therefore the results will not be representative of all Somali-born women. The findings can however inform future randomised controlled trials and be useful for future power calculations.

Conclusions

This study suggests some potential for gANC to improve information provision and knowledge acquisition among pregnant Somali-born women, with residence in Sweden <10 years. The study found few significant differences between the groups on women's overall ratings of care and emotional wellbeing measured with EPDS, and no negative impacts on either of these primary outcomes for the women who participated in gANC.

The study has demonstrated the feasibility of assessing relevant care and obstetric outcomes with Somali-born women, while noting the need for validation and possible improvement of the Somali version of the EPDS. Finally, an adequately powered randomised trial is needed for the outcomes of gANC to be robustly assessed.

Acknowledgements

We are grateful to all the women and midwives who participated and shared their experiences at different stages of this project, and to the Reference Group. We wish to commemorate midwife Ros-Mari Casselbrant who passed away in October 2019. We wish to acknowledge the following contributors specifically: research assistants Fardosa Hassen Ahmed and Aisha Adan, interpreter Hamdi Sheek Hussein and David Grannas, statistician at Biostatistics Core Facility, Karolinska Institutet.

For peer review only

For peer review only - http://bmjopen.bmj.com/site/about/guidelines.xhtml

Contributorship statement

All authors were part of the research team that developed the intervention and the questionnaires. UB and MA monitored the day-to-day implementation of the intervention and data collection at the clinic. MA and ES were responsible for the analyses. All authors have read, commented on and approved the final manuscript.

Competing interests

Conflict of interest: none to declare for any of the authors.

Funding

This work was supported by the Swedish Research Council (grant number 2015-02470), Forte (grant number 2016-00957) and the Doctoral School in Health Care Sciences, Karolinska Institutet (grant number N/A).

Data sharing statement

Deidentified participant data are available upon reasonable request from the corresponding author: Malin Ahrne, malin.ahrne@ki.se.

Ethics Approval statement

Ethical approval was obtained from Stockholm Ethical Review Board (2015/1703-31/1).

For peer review only

References

1. WHO recommendations on antenatal care for a positive pregnancy experience. World Health Organisation; 2016.
2. The Sustainable Development Agenda https://www.un.org/sustainabledevelopment/development-agenda/: United Nations; 2015 [
3. Fair F, Raben L, Watson H, Vivilaki V, van den Muijsenbergh M, Soltani H. Migrant women's experiences of pregnancy, childbirth and maternity care in European countries: A systematic review. PLoS One. 2020;15(2):e0228378.
4. Report on the health of refugees and migrants in the WHO European Region: no public health without refugee and migrant health. World Health Organization Regional Office for Europe; 2018.
5. World Migration Report 2020. International Organisation of Migration IOM; 2019.
6. Villadsen SF, Mortensen LH, Andersen AM. Care during pregnancy and childbirth for migrant women: How do we advance? Development of intervention studies--the case of the MAMAACT intervention in Denmark. Best Pract Res Clin Obstet Gynaecol. 2016;32:100-12.
7. Rogers HJ, Hogan L, Coates D, Homer CSE, Henry A. Responding to the health needs of women from migrant and refugee backgrounds-Models of maternity and postpartum care in high-income countries: A systematic scoping review. Health Soc Care Community. 2020;28(5):1343-65.
8. Liu C, Ahlberg M, Hjern A, Stephansson O. Perinatal health of refugee and asylum-seeking women in Sweden 2014-17: a register-based cohort study. Eur J Public Health. 2019;29(6):1048-55.
9. Nilsen RM, Vik ES, Rasmussen SA, Small R, Moster D Pregnancy and Childbirth. 2018;18(1):423.
10. Gagnon AJ, Zimbeck M, Zeitlin J, Alexander S, Blondel B, Buitendijk S, et al. Migration to western industrialised countries and perinatal health: a systematic review. Soc Sci Med. 2009;69(6):934-46.
11. Bastola K, Koponen P, Gissler M, Kinnunen TI. Differences in caesarean delivery and neonatal outcomes among women of migrant origin in Finland: A population-based study. Paediatr Perinat Epidemiol. 2020;34(1):12-20.
12. Bastola K, Koponen P, Härkänen T, Luoto R, Gissler M, Kinnunen TI. Delivery and its complications among women of Somali, Kurdish, and Russian origin, and women in the general population in Finland. Birth. 2019;46(1):35-41.
13. Khanolkar AR, Wedrén S, Essén B, Sparén P, Koupil I. Preterm and postterm birth in immigrant- and Swedish-born parents: a population register-based study. Eur J Epidemiol. 2015;30(5):435-47.
14. Bakken KS, Skjeldal OH, Stray-Pedersen B. Higher risk for adverse obstetric outcomes among immigrants of African and Asian descent: a comparison study at a low-risk maternity hospital in Norway. Birth. 2015;42(2):132-40.
15. Almeida LM, Caldas J, Ayres-de-Campos D, Salcedo-Barrientos D, Dias S. Maternal healthcare in migrants: a systematic review. Matern Child Health J. 2013;17(8):1346-54.
16. Belihu FB, Davey MA, Small R. Perinatal health outcomes of East African immigrant populations in Victoria, Australia: a population based study. BMC Pregnancy and Childbirth. 2016;16:86.
17. Malin M, Gissler M. Maternal care and birth outcomes among ethnic minority women in Finland. BMC Public Health. 2009;9:84.
18. Vik ES, Aasheim V, Schytt E, Small R, Moster D, Nilsen RM. Stillbirth in relation to maternal country of birth and other migration related factors: a population-based study in Norway. BMC Pregnancy and Childbirth. 2019;19(1):5.
19. Råssjö EB, Byrskog U, Samir R, Klingberg-Allvin M. Somali women's use of maternity health services and the outcome of their pregnancies: a descriptive study comparing Somali immigrants with native-born Swedish women. Sex Reprod Healthc. 2013;4(3):99-106.

For peer review only - http://bmjopen.bmj.com/site/about/guidelines.xhtml

20. Akselsson A, Lindgren H, Georgsson S, Pettersson K, Skokic V, Rådestad I. Pregnancy outcomes among women born in Somalia and Sweden giving birth in the Stockholm area - a population-based study. Global Health Action. 2020;13(1):1794107.
21. Essén B, Hanson BS, Ostergren PO, Lindquist PG, Gudmundsson S. Increased perinatal mortality among sub-Saharan immigrants in a city-population in Sweden. Acta Obstet Gynecol Scand. 2000;79(9):737-43.
22. Small R, Gagnon A, Gissler M, Zeitlin J, Bennis M, Glazier R, et al. Somali women and their pregnancy outcomes postmigration: data from six receiving countries. BJOG. 2008;115(13):1630-40.
23. Johnson EB, Reed SD, Hitti J, Batra M. Increased risk of adverse pregnancy outcome among Somali immigrants in Washington state. Am J Obstet Gynecol. 2005;193(2):475-82.
24. Oliver EA, Klebanoff M, Yossef-Salameh L, Oza-Frank R, Moosavinasab S, Reagan P, et al. Preterm Birth and Gestational Length in Four Race-Nativity Groups, Including Somali Americans. Obstet Gynecol. 2018;131(2):281-9.
25. Ahrne M, Schytt E, Andersson E, Small R, Adan A, Essén B, et al. Antenatal care for Somali-born women in Sweden: Perspectives from mothers, fathers and midwives. Midwifery. 2019;74:107-15.
26. Schmidt NC, Fargnoli V, Epiney M, Irion O. Barriers to reproductive health care for migrant women in Geneva: a qualitative study. Reprod Health. 2018;15(1):43.
27. Bains S, Skråning S, Sundby J, Vangen S, Sørbye IK, Lindskog BV. Challenges and barriers to optimal maternity care for recently migrated women - a mixed-method study in Norway. BMC Pregnancy and Childbirth. 2021;21(1):686.
28. Essén B, Johnsdotter S, Hovelius B, Gudmundsson S, Sjöberg NO, Friedman J, et al. Qualitative study of pregnancy and childbirth experiences in Somalian women resident in Sweden. BJOG. 2000;107(12):1507-12.
29. Small R, Roth C, Raval M, Shafiei T, Korfker D, Heaman M, et al. Immigrant and non-immigrant women's experiences of maternity care: a systematic and comparative review of studies in five countries. BMC Pregnancy and Childbirth. 2014;14:152.
30. Higginbottom GM, Morgan M, Alexandre M, Chiu Y, Forgeron J, Kocay D, et al. Immigrant women's experiences of maternity-care services in Canada: a systematic review using a narrative synthesis. Syst Rev. 2015;4:13.
31. Heslehurst N, Brown H, Pemu A, Coleman H, Rankin J. Perinatal health outcomes and care among asylum seekers and refugees: a systematic review of systematic reviews. BMC Med. 2018;16(1):89.
32. Heaman M, Bayrampour H, Kingston D, Blondel B, Gissler M, Roth C, et al. Migrant women's utilization of prenatal care: a systematic review. Matern Child Health J. 2013;17(5):816-36.
33. Essén B, Bödker B, Sjöberg NO, Langhoff-Roos J, Greisen G, Gudmundsson S, et al. Are some perinatal deaths in immigrant groups linked to suboptimal perinatal care services? BJOG. 2002;109(6):677-82.
34. Beine K, Fullerton J, Palinkas L, Anders B. Conceptions of prenatal care among Somali women in San Diego. J Nurse Midwifery. 1995;40(4):376-81.
35. Davies MM, Bath PA. The maternity information concerns of Somali women in the United Kingdom. J Adv Nurs. 2001;36(2):237-45.
36. Wiklund H, Aden AS, Högberg U, Wikman M, Dahlgren L. Somalis giving birth in Sweden: a challenge to culture and gender specific values and behaviours. Midwifery. 2000;16(2):105-15.
37. Herrel N, Olevitch L, DuBois DK, Terry P, Thorp D, Kind E, et al. Somali refugee women speak out about their needs for care during pregnancy and delivery. J Midwifery Women's Health. 2004;49(4):345-9.
38. Esscher A, Binder-Finnema P, Bodker B, Hogberg U, Mulic-Lutvica A, Essen B. Suboptimal care and maternal mortality among foreign-born women in Sweden: maternal death audit with application of the 'migration three delays' model. BMC Pregnancy and Childbirth. 2014;14:141.
39. Ny P, Dykes AK, Molin J, Dejin-Karlsson E. Utilisation of antenatal care by country of birth in a multi-ethnic population: a four-year community-based study in Malmö, Sweden. Acta Obstet Gynecol Scand. 2007;86(7):805-13.

For peer review only - http://bmjopen.bmj.com/site/about/guidelines.xhtml

40. Fabian H, Rådestad I, Rodriguez A, Waldenström U. Women with non-Swedish speaking background and their children: a longitudinal study of uptake of care and maternal and child health. Acta Paediatr. 2008;97(12):1721-8.
41. Fabian HM, Rådestad IJ, Waldenström U. Characteristics of Swedish women who do not attend childbirth and parenthood education classes during pregnancy. Midwifery. 2004;20(3):226-35.
42. Ludwig A, Miani C, Breckenkamp J, Sauzet O, Borde T, Doyle IM, et al. Are Social Status and Migration Background Associated with Utilization of Non-medical Antenatal Care? Analyses from Two German Studies. Matern Child Health J. 2020;24(7):943-52.
43. Catling CJ, Medley N, Foureur M, Ryan C, Leap N, Teate A, et al. Group versus conventional antenatal care for women. Cochrane Database Syst Rev. 2015;2015(2):Cd007622.
44. Carter EB, Temming LA, Akin J, Fowler S, Macones GA, Colditz GA, et al. Group Prenatal Care Compared With Traditional Prenatal Care: A Systematic Review and Meta-analysis. Obstet Gynecol. 2016;128(3):551-61.
45. Hunter L, Da Motta G, McCourt C, Wiseman O, Rayment J, Haora P, et al. `It makes sense and it works': Maternity care providers' perspectives on the feasibility of a group antenatal care model (Pregnancy Circles). Midwifery. 2018;66:56-63.
46. Cunningham SD, Lewis JB, Thomas JL, Grilo SA, Ickovics JR. Expect With Me: development and evaluation design for an innovative model of group prenatal care to improve perinatal outcomes. BMC Pregnancy and Childbirth. 2017;17(1):147.
47. Hodgson ZG, Saxell L, Christians JK. An evaluation of Interprofessional group antenatal care: a prospective comparative study. BMC pregnancy and childbirth. 2017;17(1):297.
48. Cunningham SD, Grilo S, Lewis JB, Novick G, Rising SS, Tobin JN, et al. Group Prenatal Care Attendance: Determinants and Relationship with Care Satisfaction. Matern Child Health J. 2017;21(4):770-6.
49. Liu R, Chao MT, Jostad-Laswell A, Duncan LG. Does CenteringPregnancy Group Prenatal Care Affect the Birth Experience of Underserved Women? A Mixed Methods Analysis. J Immigr Minor Health. 2017;19(2):415-22.
50. Byerley BM, Haas DM. A systematic overview of the literature regarding group prenatal care for high-risk pregnant women. BMC Pregnancy and Childbirth. 2017;17(1):329.
51. Felder JN, Epel E, Lewis JB, Cunningham SD, Tobin JN, Rising SS, et al. Depressive symptoms and gestational length among pregnant adolescents: Cluster randomized control trial of CenteringPregnancy® plus group prenatal care. J Consult Clin Psychol. 2017;85(6):574-84.
52. Hollowell J, Oakley L, Kurinczuk JJ, Brocklehurst P, Gray R. The effectiveness of antenatal care programmes to reduce infant mortality and preterm birth in socially disadvantaged and vulnerable women in high-income countries: a systematic review. BMC Pregnancy and Childbirth. 2011;11.
53. Andersson E, Christensson K, Hildingsson I. Mothers' satisfaction with group antenatal care versus individual antenatal care--a clinical trial. Sex Reprod Healthc. 2013;4(3):113-20.
54. Riggs E, Muyeen S, Brown S, Dawson W, Petschel P, Tardiff W, et al. Cultural safety and belonging for refugee background women attending group pregnancy care: An Australian qualitative study. Birth. 2017;44(2):145-52.
55. Madeira AD, Rangen CM, Avery MD. Design and Implementation of a Group Prenatal Care Model for Somali Women at a Low-Resource Health Clinic. Nurs Women's Health. 2019;23(3):224-33.
56. Statistics Sweden https://www.scb.se/hitta-statistik/sverige-i-siffror/manniskorna-i-sverige/utrikes-fodda/ 2021 [
57. Byrskog U, Ahrne M, Small R, Andersson E, Essen B, Adan A, et al. Rationale, development and feasibility of group antenatal care for immigrant women in Sweden: a study protocol for the Hooyo Project. BMJ Open. 2019;9(7):e030314.
58. (SFOG) SSoOaG. Mödrahälsovård, Sexuell och Reproduktiv Hälsa (Prenatal Care, Sexual and Repriductive Health) (Published in 2008, updated as a web-version in 2016). https://www.sfog.se/natupplaga/ARG76web4a328b70-0d76-474e-840e-31f70a89eae9.pdf; 2016.
59. Gagnon AJ, DeBruyn R, Essen B, Gissler M, Heaman M, Jeambey Z, et al. Development of the Migrant Friendly Maternity Care Questionnaire (MFMCQ) for migrants to

For peer review only - http://bmjopen.bmj.com/site/about/guidelines.xhtml

Western societies: an international Delphi consensus process. BMC Pregnancy Childbirth. 2014;14:200.
60. Cox J. Thirty years with the Edinburgh Postnatal Depression Scale: voices from the past and recommendations for the future. Br J Psychiatry. 2019;214(3):127-9.
61. Rubertsson C, Börjesson K, Berglund A, Josefsson A, Sydsjö G. The Swedish validation of Edinburgh Postnatal Depression Scale (EPDS) during pregnancy. Nord J Psychiatry. 2011;65(6):414-8.
62. Cox J HJ, Henshaw C, et al. The Edinburgh Postnatal Depression Scale (EPDS) Manual. 2 edn. : The Royal College of psychiatrists; 2012.
63. Morkved S, Bo K. Prevalence of urinary incontinence during pregnancy and postpartum. Int Urogynecol J Pelvic Floor Dysfunct. 1999;10(6):394-8.
64. PRAMS Questionnaires (Pregnancy Risk Assessment Monitoring System) Phase 8 Standard Questions February 2017 https://www.cdc.gov/prams/questionnaire.htm#current: Centers for Disease Control and Prevention (CDC); 2017 [
65. Hildingsson I, Rådestad I. Swedish women's satisfaction with medical and emotional aspects of antenatal care. J Adv Nurs. 2005;52(3):239-49.
66. Rubertsson C, Wickberg B, Gustavsson P, Rådestad I. Depressive symptoms in early pregnancy, two months and one year postpartum-prevalence and psychosocial risk factors in a national Swedish sample. Arch Women's Ment Health. 2005;8(2):97-104.
67. Gupta SK. Intention-to-treat concept: A review. Perspect Clin Res. 2011;2(3):109-12.
68. Løvlie AL, Madar AA. Postpartum Depression Among Somali Women in Norway. J Immigr Minor Health. 2017;19(3):638-44.
69. Ingvarsdotter K, Johnsdotter S, Östman M. Lost in interpretation: the use of interpreters in research on mental ill health. Int J Soc Psychiatry. 2012;58(1):34-40.
70. Renfrew MJ, McFadden A, Bastos MH, Campbell J, Channon AA, Cheung NF, et al. Midwifery and quality care: findings from a new evidence-informed framework for maternal and newborn care. Lancet. 2014;384(9948):1129-45.
71. Moore GF, Audrey S, Barker M, Bond L, Bonell C, Hardeman W, et al. Process evaluation of complex interventions: Medical Research Council guidance. BMJ. 2015;350:h1258.
72. Carter EB, Mazzoni SE. A paradigm shift to address racial inequities in perinatal healthcare. Am J Obstet Gynecol. 2021;224(4):359-61.
73. Park JH, Vincent D, Hastings-Tolsma M. Disparity in prenatal care among women of colour in the USA. Midwifery. 2007;23(1):28-37.
74. Ismail MM, Gerrish K, Naisby A, Salway S, Chowbey P. Engaging minorities in researching sensitive health topics by using a participatory approach. Nurse Res. 2014;22(2):44-8.

For peer review only

Figure legends

Figure 1. Women´s overall ratings of ANC care – the proportion of women who were "always", "mostly", "sometimes", "rarely" and "never" happy with the care they received in the two different care models, assessed in late pregnancy and two months postpartum.

Figure 2. Median scores on the Edinburgh Postnatal Depression Scale (EPDS); at baseline (Q1), late pregnancy (Q2) and 2 months postpartum (Q3) analysed by ANCOVA test of differences. The boxplot also shows minimum and maximum score, and the 1st and 3rd quartile and the mean differences with 95% CI at each time point.

For peer review only

For peer review only - http://bmjopen.bmj.com/site/about/guidelines.xhtml

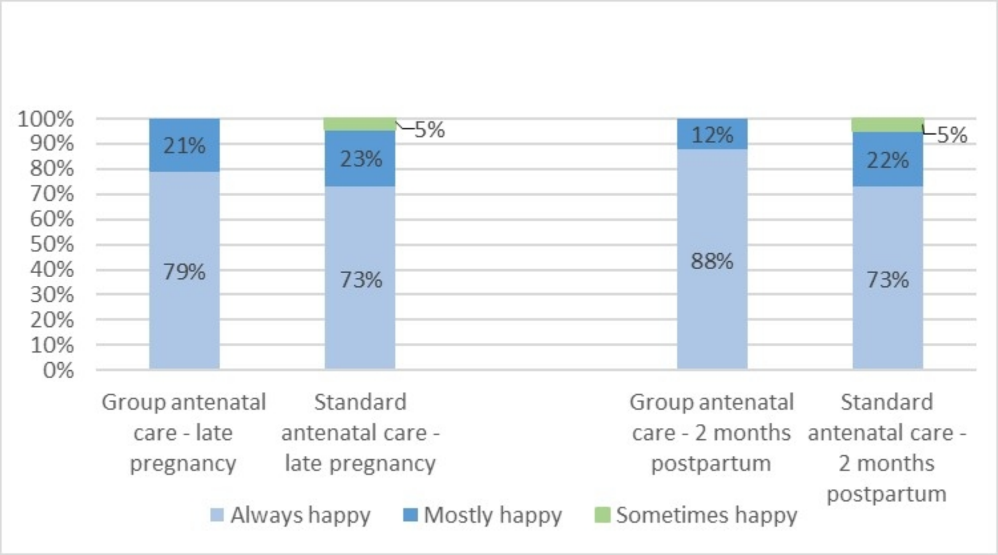

Figure 1. Women´s overall ratings of ANC care – the proportion of women who were "always", "mostly", "sometimes", "rarely" and "never" happy with the care they received in the two different care models, assessed in late pregnancy and two months postpartum.

127x70mm (150 x 150 DPI)

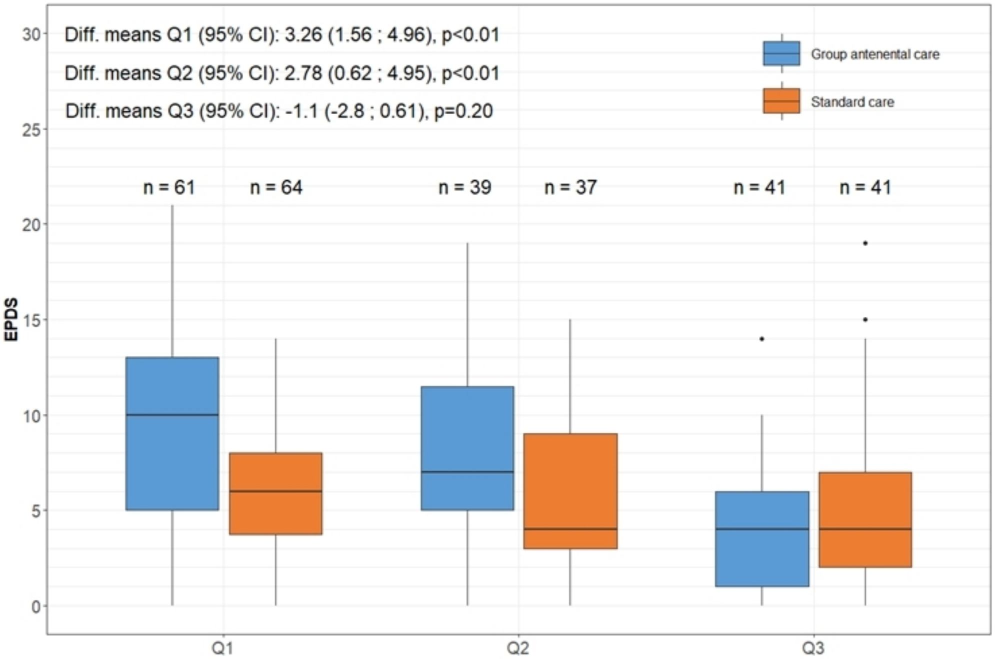

Figure 2. Median scores on the Edinburgh Postnatal Depression Scale (EPDS); at baseline (Q1), late pregnancy (Q2) and 2 months postpartum (Q3) analysed by ANCOVA test of differences. The boxplot also shows minimum and maximum score, and the 1st and 3rd quartile and the mean differences with 95% CI at each time point.

125x88mm (150 x 150 DPI)

BMJ Open

Group antenatal care compared with standard antenatal care for Somali-Swedish women: a historically controlled evaluation of the Hooyo-project

Journal:	*BMJ Open*
Manuscript ID	bmjopen-2022-066000.R1
Article Type:	Original research
Date Submitted by the Author:	19-Dec-2022
Complete List of Authors:	Ahrne, Malin ; Karolinska Institutet, Department of Women's and Children's Health<br>Byrskog, Ulrika; Dalarna University School of Education Health and Social Studies<br>Essen, Birgitta; Uppsala University, Women's and Children's Health<br>Andersson, Ewa; Karolinska Institutet, Department of Women's and Children's Health<br>Small, Rhonda; Karolinska Institutet, Department of Women's and Children's Health; La Trobe University<br>Schytt, Erica; Uppsala University Center for Clinical Research Dalarna; Western Norway University of Applied Sciences
**Primary Subject Heading**:	Obstetrics and gynaecology
Secondary Subject Heading:	Reproductive medicine
Keywords:	Antenatal < GENETICS, Prenatal diagnosis < OBSTETRICS, Maternal medicine < OBSTETRICS, Reproductive medicine < GYNAECOLOGY, SOCIAL MEDICINE

SCHOLARONE™
Manuscripts

I, the Submitting Author has the right to grant and does grant on behalf of all authors of the Work (as defined in the below author licence), an exclusive licence and/or a non-exclusive licence for contributions from authors who are: i) UK Crown employees; ii) where BMJ has agreed a CC-BY licence shall apply, and/or iii) in accordance with the terms applicable for US Federal Government officers or employees acting as part of their official duties; on a worldwide, perpetual, irrevocable, royalty-free basis to BMJ Publishing Group Ltd ("BMJ") its licensees and where the relevant Journal is co-owned by BMJ to the co-owners of the Journal, to publish the Work in this journal and any other BMJ products and to exploit all rights, as set out in our licence.

The Submitting Author accepts and understands that any supply made under these terms is made by BMJ to the Submitting Author unless you are acting as an employee on behalf of your employer or a postgraduate student of an affiliated institution which is paying any applicable article publishing charge ("APC") for Open Access articles. Where the Submitting Author wishes to make the Work available on an Open Access basis (and intends to pay the relevant APC), the terms of reuse of such Open Access shall be governed by a Creative Commons licence – details of these licences and which Creative Commons licence will apply to this Work are set out in our licence referred to above.

Other than as permitted in any relevant BMJ Author's Self Archiving Policies, I confirm this Work has not been accepted for publication elsewhere, is not being considered for publication elsewhere and does not duplicate material already published. I confirm all authors consent to publication of this Work and authorise the granting of this licence.

For peer review only

Group antenatal care compared with standard antenatal care for Somali-Swedish women: a historically controlled evaluation of the Hooyo-project

Malin Ahrne[a], Ulrika Byrskog[b], Birgitta Essén[c], Ewa Andersson[a], Rhonda Small[ad], Erica Schytt[ef]

Affiliations:
[a]Department of Women's and Children's Health, Karolinska Institutet, Stockholm, Sweden
[b]School of Health and Welfare, Dalarna University, Falun, Sweden
[c]Women's and Children's Health, IMCH, Uppsala University, Sweden
[d]Judith Lumley Centre, La Trobe University, Melbourne, Australia
[e]Centre for Clinical Research Dalarna - Uppsala University, Falun, Sweden
[f]Faculty of Health and Social Sciences, Western Norway University of Applied Sciences, Norway

Email addresses for authors:

malin.ahrne@ki.se (M. Ahrne), uby@du.se (U. Byrskog), birgitta.essen@kbh.uu.se (B. Essén), Ewa.Andersson@ki.se (E. Andersson), R.Small@latrobe.edu.au (R. Small), Erica.Schytt@regiondalarna.se (E. Schytt)

Correspondence to:
Malin Ahrne
Department of Women´s and Children´s Health
Karolinska Institutet
Tomtebodavägen 18A
SE-171 77 Stockholm, Sweden
E-mail: malin.ahrne@ki.se

For peer review only

Licence Statement

I, the Submitting Author has the right to grant and does grant on behalf of all authors of the Work (as defined in the below author licence), an exclusive licence and/or a non-exclusive licence for contributions from authors who are: i) UK Crown employees; ii) where BMJ has agreed a CC-BY licence shall apply, and/or iii) in accordance with the terms applicable for US Federal Government officers or employees acting as part of their official duties; on a worldwide, perpetual, irrevocable, royalty-free basis to BMJ Publishing Group Ltd ("BMJ") its licensees and where the relevant Journal is co-owned by BMJ to the co-owners of the

Journal, to publish the Work in BMJ Open and any other BMJ products and to exploit all rights, as set out in our licence.

The Submitting Author accepts and understands that any supply made under these terms is made by BMJ to the Submitting Author unless you are acting as an employee on behalf of your employer or a postgraduate student of an affiliated institution which is paying any applicable article publishing charge ("APC") for Open Access articles. Where the Submitting Author wishes to make the Work available on an Open Access basis (and intends to pay the relevant APC), the terms of reuse of such Open Access shall be governed by a Creative Commons licence – details of these licences and which Creative Commons licence will apply to this Work are set out in our licence referred to above.

For peer review only

Abstract

Objectives: Comparing language-supported group antenatal care (gANC) and standard antenatal care (sANC) for Somali-born women in Sweden, measuring overall ratings of care and emotional wellbeing, and testing the feasibility of the outcome measures.

Design: A quasi-experimental trial with one intervention and one historical control group, nested in an intervention development and feasibility study**.**

Setting: Midwifery-led antenatal care clinic in a mid-sized Swedish town.

Participants: Pregnant Somali-born women (<25 gestational weeks); 64 women in gANC and 81 in sANC.

Intervention: Language-supported gANC (2017-2019). Participants were offered seven 60-minute group-sessions with other Somali-born women led by 1-2 midwives, in addition to 15-30 minute individual appointments with their designated midwife.

Outcomes: Primary outcomes were women's overall ratings of antenatal care and emotional wellbeing (Edinburgh Postnatal Depression Scale (EPDS)) in gestational week ≥35 and 2-months postpartum. Secondary outcomes were specific care experiences, information received, social support, knowledge of pregnancy danger signs and obstetric outcomes.

Results: Recruitment and retention of participants was challenging. Of eligible women, 39.3% (n=106) declined to participate. No relevant differences regarding overall ratings of antenatal care between the groups were detected (late pregnancy OR 1.42, CI 95% 0.50-4.16 and 6-8 weeks postpartum OR 2.71, CI 95% 0.88-9.41). The reduction in mean EPDS-score was greater in the intervention group when adjusting for differences at baseline (mean difference -1.89; CI 95% -3.73 - -0.07). Women in gANC were happier with received pregnancy and birth information, e.g. caesarean section where 94.9% (n=37) believed the information was sufficient compared to 17.5% (n=7) in standard care (p<0.001) in late pregnancy.

Conclusions: This evaluation suggests potential for language-supported gANC to improve knowledge acquisition among pregnant Somali-born women with residence in Sweden <10 years. An adequately powered randomised trial is needed to evaluate the effectiveness of the intervention.

For peer review only - http://bmjopen.bmj.com/site/about/guidelines.xhtml

Strengths and limitations of this study

- This study was part of an intervention development and feasibility study powered to detect clinically relevant differences in women's overall ratings of antenatal care and the Edinburgh Postnatal Depression Scale (EPDS) but not its impact in relation to other outcomes.
- The study had a participatory approach that integrated the perspectives of midwives and Somali-born women in the intervention development and in the study design.
- The recruitment goal of 63 women in each group was reached and therefore the study had power to detect differences between the groups in emotional wellbeing measured with the Edinburgh Postnatal Depression Scale but not overall ratings of care.
- Emotional wellbeing measured with the Edinburgh Postnatal Depression Scale contributes to the evidence for its use, as it is not yet validated for Somali-speaking women.
- Women were recruited from a single site only, and possible selection bias may further limit the representativeness and generalisability of the results; however, the findings may be useful for future power calculations.

For peer review only

Introduction

To improve maternal health in Sweden and globally, there is a need for innovative evidence-based approaches to antenatal care (1-3). Migration interacts with other social health determinants contributing to health inequalities (4, 5). With a changing demography of pregnant women in many high-income countries, rethinking how care is provided to migrant women is necessary to improve pregnancy outcomes (3, 6, 7). Studies show that many migrant women in high-income countries are at an increased risk of poor pregnancy outcomes such as higher incidence of co-morbidities (8), pre-eclampsia (9), preterm birth (10-12), post-term birth (13, 14), lower use of some obstetrical interventions (15), higher risk of emergency caesarean birth, postpartum depression and maternal death (15), and for infants, higher risk of low Apgar score (11, 14), low birthweight (8, 11, 14, 16, 17), small for gestational age (SGA) [illegible]

For Somali-born women, higher rates of complications during pregnancy and birth compared to the general population of migrant receiving countries have been reported, such as anaemia and severe hyperemesis (19), fetal distress, perineal laceration, postpartum haemorrhage (12), higher incidence of caesarean sections, SGA (19, 20) perinatal deaths (21) and stillbirths (22, 23). A lower rate of preterm birth in Somali-born women has been observed (16, 22, 24), and [illegible]

Qualitative studies (25-28) and systematic reviews show that migrant women more often report poor experiences of maternity care, such as communication problems (3, 29-31), lack of familiarity with care systems (3, 29, 30), sub-optimal care (3, 15, 30, 32) and discrimination (3, 15, 29, 31). The risk of maternal and reproductive health inequalities and sub-optimal care affecting Somali-born women has been described for decades (23, 33-38). Swedish studies show that migrant women (39), including Somali-born women (19), commence ANC later, make fewer ANC visits and are less likely to contact obstetric care for decreased fetal movements (20). Finally, lower attendance in childbirth preparation and parenting classes during pregnancy among migrant women in Sweden and elsewhere has also been reported (40-42).

New models of maternity care that address migrant women's specific socioeconomic and psychosocial circumstances have been proposed (3). One such model is group antenatal care (gANC), which typically incorporates pregnancy check-ups and group sessions for education

For peer review only - http://bmjopen.bmj.com/site/about/guidelines.xhtml

and social support in a group of women at similar stages of pregnancy (43, 44). Slightly different models of gANC have been developed in various settings (45-47). gANC has the potential to increase ANC attendance, improve satisfaction with care and pregnancy outcomes such as lower rates of preterm birth, increased breastfeeding rates and reduced risk of depressive symptoms (48-51), despite some inconsistency in the evidence (44, 47, 52). In a Swedish setting, low-risk women in gANC were more satisfied with the information received about labour and birth, and with the midwives´ engagement (53), however women with inadequate Swedish-proficiency were not included. Studies of gANC for migrant women are scarce, but there are some promising examples of small-scale interventions (7, 54, 55). None has been conducted in Sweden, where 26% of the population is foreign-born (56).

The aim of this study was to compare language-supported group antenatal care and standard antenatal care for Somali-born women in Sweden in terms of women's overall ratings of care and their emotional wellbeing in late pregnancy and 2-months postpartum, and to evaluate the feasibility of the outcome measures.

For peer review only

For peer review only - http://bmjopen.bmj.com/site/about/guidelines.xhtml

Methods

Study design

A single-site, quasi-experimental study with one intervention and one historical control group was conducted between October 2016 and September 2019, as part of the Hooyo-project (‘mother’ in Somali), a development and feasibility study of gANC for Somali-born women in Sweden. The intervention group received gANC (May 2017 – September 2019) and the control group received standard antenatal care (sANC) (October 2016 – May 2017). Questionnaires were used at three time points to assess ratings of care, emotional wellbeing and a number of secondary outcomes. Additional data were retrieved from patient records. The study was registered in ClinicalTrials.gov (Identifier: NCT03879200).

Patient and public involvement

The intervention was developed in collaboration with midwives and members of the Somali community; the process is described in detail elsewhere (57). Initial focus group discussions with Somali women and men informed the intervention design (25). A study reference group included midwives and members of the Somali community with relevant professional backgrounds and was engaged in all stages of the intervention development. A bicultural research assistant was responsible for recruiting women to the study and conducting the interviews.

Participants and recruitment

Participants were pregnant (<25 gestational weeks (gwk)) Somali-born women. Exclusion criteria were severe health conditions (e.g. need of specialist obstetric care or a severe mental health condition). At the first ANC appointment, midwives provided initial oral and written information to all eligible women and if agreed, the bilingual research assistant then provided in-depth information, recruited those interested to the study and obtained consent. Information materials were available in Swedish and Somali.

Setting

Two ANC clinics were involved in developing the model and one clinic implemented the intervention. The implementing clinic is public and located in a mid-sized town in Sweden, with a mixed socioeconomic uptake area, and has 10 midwives.

For peer review only - http://bmjopen.bmj.com/site/about/guidelines.xhtml

Antenatal care in Sweden is free of charge with continuity of care throughout pregnancy, and referral to an obstetrician or other specialist when needed. A minimum of 8-9 midwife appointments is recommended (58), exclusive of ultrasound for pregnancy dating, which is recommended in gwk 18-20. Appointments are usually 30 minutes. A first, early visit focuses on lifestyle factors. The second visit is usually 45 minutes, including a detailed patient history. Visits 3-9 include health controls of the woman and unborn baby and provision of information, including information about danger signs like vaginal bleeding, leakage of amniotic fluids, decreased fetal movement and symptoms of preeclampsia and where to seek healthcare. Language interpretation can be arranged, either telephone or face to face. ANC clinics offer additional birth preparation in groups and parenting classes. Partners are welcome to attend both individual appointments and classes.

The Hooyo gANC-intervention

The intervention was a combination of group antenatal care and individual check-ups, with language support and integrated childbirth and parenting education. Participants were offered seven 60-minute group sessions together with other women of similar gestational age, led by 1-2 midwives. Group rules were set by participants, so partners were mostly but not always invited to participate. Directly before or after each group session, a 15-minute individual appointment was scheduled for health check-ups with the designated midwife. Participants started attending group antenatal care in gwk 20-26. Additional individual appointments were scheduled if needed. Frequency and total number of appointments followed the Swedish national ANC recommendations (58). The groups were fixed, meaning that women were assigned to a specific group with the same participants and a specific start and end date. Typically, the sessions started with a presentation of a selected topic, following Swedish national guidelines; life style, pregnancy, birth, practical birth preparations, the new-born baby, breast feeding (and alternatives), parenthood and relationships (58). The midwives were to be responsive to the interests and concerns of the group, and to encourage questions, dialogue etc. Language support was provided by a female interpreter in every group session, usually the same interpreter who was also a trained nurse assistant. The interpreter also served as a co-facilitator of the group. Before start-up, the midwives received 1 ½ - days training on person-centred care, use of motivational interviewing in groups and group dynamics. A manual was developed. The midwives planned the sessions and chose relevant pedagogical tools like films or anatomical models.

For peer review only

Control group

The control group received standard, midwifery-led individual care in accordance with Swedish national guidelines (58) as described above.

Data collection

Three questionnaires with both closed and open-ended questions were developed by the research team in English and translated to Swedish and Somali and are described in detail in the study protocol (57). Some questions from the Migrant Friendly Maternity Care Questionnaire (MFMCQ) were included but adapted slightly (59). The Edinburgh Postnatal Depression Scale (EPDS) was included in all three questionnaires (see below) (60).

A bilingual research assistant recorded women's responses to the questionnaires during face-to-face or telephone interviews; questionnaire one (Q1) in gwk 21-25, the second questionnaire (Q2) in late pregnancy (<gwk 35) and the third questionnaire (Q3) for the follow-up at two months postpartum.

Baseline information on women's health, such as age, obstetric history, height, weight, use of tobacco in early pregnancy, diabetes mellitus type 2 (ICD code O24.1), haemoglobin and S-ferritin were retrieved from patient records. Gestational age at first ANC visit is presented in intervals ≤12; 13-20 and 21-25 gwk (after which women were not eligible). Ultrasound for dating of pregnancy is recommended in Sweden in gwk 18-20 (58), making week 20 a reasonable upper limit for the second interval. The questionnaires included sociodemographic information (i.e., language proficiency, level of education, marital status, occupational and migration factors). Entire household monthly disposable income was self-reported in Swedish kronor (SEK). Household size is the self-reported number of persons living in the same household.

Primary outcomes

Women's overall rating of antenatal care was assessed in late pregnancy and 2-months postpartum with the core question 'When thinking about your overall experience of antenatal care — in general, have you been happy with the care that you have received?' with response alternatives *always, mostly* (happy with care) *and sometimes, rarely* and *never* (not happy with care).

For peer review only - http://bmjopen.bmj.com/site/about/guidelines.xhtml

The EPDS is a 10-item scale initially developed to screen for postnatal depression symptoms, which is validated for use in Swedish during pregnancy (61) and has been translated to Somali but not validated (62). The validated language versions of the EPDS are routinely used for screening of mothers postpartum in child health units in Sweden (63). The 10 items are scored 0-3 according to severity of the self-reported symptoms, with a maximum score of 30. The scale gives an indication of depressive symptoms over the last 7 days. In Sweden, ≥13 points has been validated as an optimal cut-off for detecting depression in pregnant women (61). The EPDS was used at baseline, in late pregnancy and postpartum.

Secondary outcomes

Women's ratings of different components of antenatal care in late pregnancy and 2-months postpartum were also assessed. A number of questions in this format were asked: *"Have you been happy with…?"* with response alternatives *always, mostly, sometimes, rarely* and *never*. Responses were dichotomised in two categories: *"always + mostly"* and "*sometimes + rarely + never"*). Women's ratings of receiving sufficient information about pregnancy, labour and birth as well as social support were assessed (response alternatives *yes* or *no)* in late pregnancy and two months postpartum. Social support during pregnancy was measured by modified questions from the Pregnancy Risk Assessment Monitoring System (PRAMS) (64). The Cambridge Worry Scale (65) was included in the questionnaires but responses were not analysed because of overlap with other questions related to worries.

Knowledge about danger signs (vaginal bleeding, leakage of amniotic fluids, decreased fetal movement, and severe headache) and where to seek healthcare with specific symptoms (General Practitioner, labour ward, emergency room etc.) were assessed in Q2, in the question format *"What would you do if you experienced…."* with the following response alternatives *wait/self-care; contact the ANC midwife; general practitioner (GP) or nurse; delivery ward or emergency ward*.

Obstetric outcomes included: antenatal care parameters (number of ANC visits, number of visits to specialist care, referral to an obstetrician, asked about experience of violence, attendance at parent education), health parameters (Haemoglobin (lowest value and last value prior to birth), S-Ferritin (lowest value), weight gain during pregnancy, gestational diabetes mellitus (ICD-10 code O24.4)), birth outcomes (induction of labour, oxytocin for dystocia,

For peer review only - http://bmjopen.bmj.com/site/about/guidelines.xhtml

pain relief, mode of birth, perineal injury, blood loss, breast feeding at the labour ward, length of stay), attendance at postpartum check-up, breastfeeding and Body Mass Index (BMI) at the postpartum visit. The number of ANC visits is presented as the median number of visits, and the proportion of women having 6 or fewer visits, 7-11 visits, or more than 11 visits. This was considered more clinically relevant to the Swedish context than using the Adequacy of Prenatal Care Utilization Index (APCUI) as originally planned and stated in the study protocol (57).

Infant outcomes: gestational age, birthweight, SGA (ICD-10 codes P05.0 and P05.1), large for gestational age (LGA) (ICD-10 codes P08.0 and P08.1), Apgar score <7 at five minutes, umbilical cord pH (arterial and venous) and neonatal intensive care.

Sample size calculation

The study was designed to have power to detect clinically relevant differences in women's overall ratings of antenatal care and a difference in means on the EPDS. The initial sample size calculation of 70 women in each group (with 80% power and an alpha of 20%) was based on a national population study on Swedish speaking women's satisfaction with ANC two months postpartum (66). Our assumption was that the ratings of care would improve, from 65% of women receiving individual care being happy with the ANC care received (*"always + mostly happy with care"*) to 82% of those receiving gANC.

To have similar power to detect differences in EPDS mean scores, 63 women were required in each group, based on a hypothesized reduction from a mean of 8.0 in the control group to that of 6.0 in the intervention group (67). To allow for loss to follow-up with 20%, a total of 174 women needed to be recruited.

Statistical analyses

Women who were recruited to the intervention and control groups and contributed data were analysed according to the intention-to-treat (ITT) concept (68). Frequencies and percentages are reported for dichotomous variables, median and interquartile ranges (IQR) are reported for continuous variables. Chi-square tests were performed to test hypotheses for dichotomous variables, Mann-Whitney U tests for continuous variables. For the primary outcome "overall ratings of care" odds ratios (OR) with 95% confidence intervals (CI) were calculated.

For peer review only - http://bmjopen.bmj.com/site/about/guidelines.xhtml

An ANCOVA test was performed to test the difference in mean EPDS scores between gANC and standard care at two months postpartum, adjusted for differences in EPDS at baseline, after the exclusion of women with missing EPDS values (gANC n=41, sANC n=38). P-values <0.05 were deemed statistically significant and all tests were two tailed. All statistical analyses were performed in R version 4.01.

Results

Of 270 eligible women, 145 women were recruited to the study (53.7%) – 64 women to gANC and 81 to sANC. The number of women who declined to participate was 106 (39.3% of all eligible women), and 19 women did not meet the inclusion criteria (n=6) or were excluded for other reasons (n=13), such as moving or having a miscarriage.

The first questionnaire (Q1) was completed by 129 women (89% of all women recruited to the study) (gANC n=62; sANC n=67); the second questionnaire (Q2) by 80 women (55%) (gANC n=40; sANC n=40) and the third (Q3) by 86 women (59%) (gANC n=44; sANC n=42). Of the women in gANC and sANC, 38 women (59.4%) and 32 women (39.5%) responded to all three questionnaires respectively.

Baseline characteristics for the sample are described in Table 1. The groups were largely similar, however, a larger proportion of women in gANC had less than six years of education, spoke Somali well or fluently, had no previous births in Sweden and were involved in home duties, on parental leave or were unemployed. The median length of residence in Sweden in this sample was seven years.

Table 1. Characteristics of the sample

	Group antenatal care (n=63)		Standard antenatal care (n=73)	
	n (%)	Median; (IQR)	n (%)	Median; (IQR)
**Sociodemographics**				
Age (years)		31 (26.3; 33.8)		30 (25.0; 33.3)
Language proficiency, well/fluent				
Swedish	41 (65.1)		41 (56.2)	
Somali	62 (98.4)		60 (82.2)	
English	15 (23.8)		14 (19.2)	
Missing	1 (1.6)		8 (11.0)	
Completed level of education				
<6 years	32 (50.8)		25 (34.2)	
7-9 years	14 (22.2)		18 (24.7)	

For peer review only - http://bmjopen.bmj.com/site/about/guidelines.xhtml

>10 years	14 (22.2)		13 (17.8)	
Missing	3 (4.8)		17 (23.3)	
Marital status				
Married/engaged/in a relationship	58 (92.1)		66 (90.4)	
Divorced/widowed/single	4 (6.3)		0 (0.0)	
Missing	1 (1.6)		7 (9.6)	
Living with husband or partner	49 (78.8)		55 (75.3)	
Missing	14 (22.2)		18 (24.7)	
Household size		3 (2.0; 5.0)		4 (3.0; 6.0)
Occupation				
Employed	12 (19.0)		12 (16.4)	
Student	19 (30.2)		27 (37.0)	
Home duties/parental leave/unemployed	30 (47.6)		23 (31.5)	
Missing	2 (3.2)		11 (15.1)	
Entire household monthly disposable income (SEK)		19,000 (13,000; 31,750)		22,000 (19,000; 28,000)
Migration				
Length of residence (years)		7 (5.0; 8.8)		7 (4.0; 8.0)
Reason for migration				
Refugee/asylum seeker	32 (50.8)		30 (41.1)	
Family ties	30 (47.6)		34 (46.6)	
Other	0 (0.0)		1 (1.4)	
Missing	1 (1.6)		8 (11.0)	
Current migration status				
Asylum seeker	4 (4)		1 (1.4)	
Permanent residency	35 (55.6)		42 (57.5)	
Swedish citizen	23 (36.5)		22 (30.1)	
Missing	1 (1.6)		8 (11.0)	
**Obstetric history**				
Parity				
0	11 (17.5)		14 (19.2)	
1-2	18 (28.6)		18 (24.7)	
3-4	18 (28.6)		17 (23.3)	
≥5	15 (23.8)		18 (24.7)	
Missing	1 (1.6)		6 (8.1)	
Previous stillbirths/neonatal deaths	4 (6.5)		4 (5.8)	
No previous birth in Sweden	5 (7.9)		0 (0.0)	
**Current pregnancy**				
Gestational age (weeks) at first ANC visit		11 (8.8; 14.0)		12 (10.0; 14.0)
≤12	32 (51.6)		38 (55.1)	
13-20	14 (22.6)		21 (30.4)	
≥ 21 - 25 (after that not eligible)	2 (3.2)		1 (1.4)	
Missing	14 (22.6)		9 (13.0)	
Body Mass Index (kg/m²) at first visit		27.8 (24.3; 31.4)		27.6 (24.7; 31.3)
Underweight (<18.5)	3 (4.8)		4 (5.8)	
Normal weight (18.5-24.9)	14 (22.6)		16 (23.2)	
Overweight (25.0-29.9)	17 (27.4)		23 (33.3)	
Obesity (≥30)	20 (32.3)		22 (31.9)	
Missing	8 (12.9)		4 (5.8)	
Tobacco use in early pregnancy	0		0	

For peer review only - http://bmjopen.bmj.com/site/about/guidelines.xhtml

S-Ferritin at first visit		23.5 (15.8; 54.0)		23.5 (13.8; 42.8)
S-Haemoglobin at first visit		121.0 (113.3; 124.8)		121.5 (117.3; 126.8)
Diabetes mellitus type 2 prior to pregnancy	1 (1.6)		1 (1.4)	

Overall ratings of care

Women's overall rating of antenatal care was assessed through the core question *'When thinking about your overall experience of antenatal care in general, have you been happy with the care that you have received?'* in late pregnancy and 2-months postpartum, with the response alternatives *always, mostly*, *sometimes, rarely* and *never*. The vast majority of women in both groups responded *always* and some responded *mostly.* Very few women (5%), and only in the control group, responded *sometimes*, and there were no responses in the categories *rarely* and *never* (Figure 1). When the variable was dichotomised following the protocol, all women (100%) in the gANC group were happy (*always* + *mostly*) with the care they had received when asked in late pregnancy, compared to 96% in the control group (sANC). Similarly, two months postpartum, all (100%) of women who participated in gANC were happy with the care they had received, compared to 95% of women in sANC (statistical test not applicable). There were no statistical differences between the groups at either time point when the outcome measure was dichotomised into *always* versus *all other alternatives* (late pregnancy OR 1.42, CI 95% 0.50-4.16 and 6-8 weeks postpartum OR 2.71, CI 95% 0.88-9.41).

Emotional wellbeing

At baseline, 61 (98.4%) women in gANC and 64 (95.5%) women in sANC responded to all ten EPDS questions. In late pregnancy, 39 (97.5%) women in gANC and 37 (92.5%) women in sANC responded, and at the last measurement 2-months postpartum, 41 women in both groups respectively responded to all items (gANC 93.2%; sANC 97.6%).

Mean EPDS scores were higher in women in gANC than in women in sANC at baseline (gANC 9.19; sANC 5.94; diff in means 3.26; CI 95% 1.56-4.96) and in late pregnancy (gANC 8.51; sANC 5.73; diff means 2.78; CI 95% 0.62-4.95) (Figure 2). Two months postpartum, the mean EPDS score was similar between the groups (gANC 3.90; sANC 5.00; diff means -1.1; CI 95% -2.80-0.61). An ANCOVA test of differences in the reduction of mean values (adjusted for differences at baseline) was made for women who responded to all three questionnaires (gANC n=41; sANC n=38). The reduction in mean EPDS-score was

For peer review only

For peer review only - http://bmjopen.bmj.com/site/about/guidelines.xhtml

greater in the intervention group when adjusting for differences at baseline (mean difference -1.89; CI 95% -3.73 – -0.07).

We also checked the mean EPDS score of women who only responded to Q1. In women who later dropped out of the study, the mean EPDS score at baseline was 7.2 (median 6.0; IQR 4.0-10.0), which was somewhat lower than the EPDS scores of all women (mean 7.5, median 7.0; IQR 4.0-11.0).

Secondary outcomes

Ratings of care

Women's ratings of specific components of antenatal care in relation to care model in late pregnancy and two months postpartum are presented in Table 2. Few statistically significant differences could be detected between the groups. Women in gANC were slightly happier with the advice they had received about how to manage common pregnancy disorders and they were more likely to report that the midwives had been encouraging and supportive (Table 2). No woman reported experience of negative discrimination by the midwives based on ethnicity, religion, culture, language etc.

Women's ratings of care during labour and birth as well as during the postpartum stay were also assessed at two months postpartum, and no differences between the groups were detected. Higher proportions of women in gANC, were *always or mostly happy with care* compared with women in sANC, both during labour and birth (gANC 60.3% (n=38); sANC 49.3% (n=36); p=0.423) and on the postpartum ward (gANC 63.5% (n=40); sANC 50.7% (n=37); p=0.218) though neither difference was statistically significant (not presented in table).

Table 2. Women's ratings of satisfaction with specific components of antenatal care (*"always or mostly happy with care"*) in relation to care model in late pregnancy and two months postpartum

	Late pregnancy			Two months postpartum		
	Group antenatal care n (%)	Standard antenatal care n (%)	p-value	Group antenatal care n (%)	Standard antenatal care n (%)	p-value
Specifically, were you happy with…						
how the health of the baby was monitored during pregnancy?	40 (100.0)	38 (95.0)	0.990	44 (100.0)	42 (100.0)	NA
how your own health was monitored during pregnancy?	40 (100.0)	37 (94.9)	0.463	44 (100.0)	40 (95.2)	0.454
the management of your physical health?[1]	40 (100.0)	35 (89.7)	0.117			
the support you received to care for your own health? (i.e. life style factors)	39 (97.5)	32 (82.1)	0.057	42 (95.5)	38 (90.5)	0.629

For peer review only - http://bmjopen.bmj.com/site/about/guidelines.xhtml

the advice you received to manage common pregnancy disorders?	37 (92.5)	29 (74.4)	0.061	44 (100.0)	34 (81.0)	0.008
Did the ANC midwives…						
listen to your own concerns?	40 (100.0)	38 (100.0)	NA	44 (100.0)	40 (95.2)	0.454
make your partner feel welcomed and included?	37 (92.5)	34 (97.1)	1.000	40 (93.0)	39 (92.9)	1.000
show respect?[1]	39 (97.5)	37 (94.9)	0.982			
make you feel welcomed?[1]	39 (97.5)	39 (100.0)	1.000			
make you feel negatively discriminated against (because of ethnicity, religion, culture, language, etc.)?[1]	0 (0.0)	0 (0.0)				
have enough time for you during your visits?[1]	40 (100.0)	39 (100.0)	NA			
take your worries seriously?[1]	40 (100.0)	34 (91.9)	0.212			
keep you informed about the progress of your pregnancy?[1]	40 (100.0)	38 (97.4)	0.990			
been encouraging and supportive?[1]	40 (100.0)	32 (82.1)	0.016			
Did you feel comfortable to ask questions?[1]	40 (100.0)	39 (100.0)	NA			
Did you understand the information that you received from the midwives?[1]	40 (100.0)	38 (100.0)	NA			
Do you think that that the information you received from the midwives was relevant?[1]	39 (100.0)	37 (92.5)	0.248			

[1] Only in questionnaire 1.

Sufficient information and social support

Both in late pregnancy and two months postpartum, women in gANC were generally happier with the information received on different aspects of pregnancy, labour and birth, breastfeeding, the care of the newborn baby and physical and emotional changes. Far more women in gANC were happy with information about caesarean section (gANC 94.9%; sANC 17.5%; p<0.001), on the management of FGM/C (gANC 79.5%; sANC 40.0%; p=0.001) and the father's/partner's role during labour and birth (gANC 94.6%; sANC 42.5%; p<0.001) (Table 3) than women in sANC.

Table 3 also shows how women viewed the social support they received. No differences between the groups were observed when assessed in late pregnancy. At two months postpartum, 97.6% of women who had attended gANC reported that they had someone who could help with a temporary place to live if they should need it, compared to 25.0% of women in standard ANC (p<0.001). Additionally, 95.2% in gANC reported that they had made new friends through the ANC clinic during pregnancy, compared to 43.9% of women in standard care (p<0.001).

For peer review only

Table 3. Sufficient information and social support comparing gANC with sANC care

	Late pregnancy			Two months postpartum		
	Group antenatal care n (%)	Standard antenatal care n (%)	p-value	Group antenatal care n (%)	Standard antenatal care n (%)	p-value
**Sufficient information**						
Ratings for having received enough information about (yes vs no)						
why it is important to take iron tablets	39 (100.0)	38 (95.0)	0.368	43 (97.7)	39 (92.9)	0.576
when to go to hospital for labour	39 (100.0)	27 (67.5)	<0.001	44 (100.0)	37 (90.2)	0.107
management of FGM/C	31 (79.5)	16 (40.0)	0.001	39 (88.6)	21 (51.2)	<0.001
management of a delivery	36 (94.7)	17 (42.5)	<0.001	44 (100.0)	27 (64.3)	<0.001
pain relief during labour	38 (97.4)	12 (30.0)	<0.001	44 (100.0)	29 (69.0)	<0.001
induction of labour	33 (84.6)	11 (27.5)	<0.001	42 (95.5)	25 (59.5)	<0.001
vacuum extraction	33 (84.6)	6 (15.0)	<0.001	41 (93.2)	19 (45.2)	<0.001
caesarean section	37 (94.9)	7 (17.5)	<0.001	43 (97.7)	22 (52.4)	<0.001
father's/partner's role during labour	35 (94.6)	17 (42.5)	<0.001	43 (97.7)	26 (61.9)	<0.001
care at the neonatal care unit	38 (97.4)	21 (52.5)	<0.001	43 (97.7)	31 (73.8)	0.004
breastfeeding	38 (97.4)	23 (57.5)	<0.001	44 (100.0)	33 (78.6)	0.004
care of the newborn baby	38 (60.3)	21 (28.8)	<0.001	42 (97.7)	34 (81.0)	0.031
care for you after labour/who to contact after discharge	37 (94.9)	15 (37.5)	<0.001	44 (100.0)	29 (72.5)	0.001
physical changes during pregnancy[1]	39 (100.0)	30 (75.0)	0.004			
emotional changes during pregnancy[1]	37 (94.9)	29 (72.5)	0.001			
taking care of your own health during pregnancy?[1]	39 (100.0)	35 (87.5)	0.074			
when to contact the midwife or hospital between the scheduled ANC visits[1]	39 (100.0)	32 (80.0)	0.013			
physical changes after pregnancy[2]				44 (100.0)	29 (69.0)	<0.001
emotional changes after pregnancy[2]				42 (95.5)	24 (57.1)	<0.001
taking care of your own health after pregnancy[2]				43 (97.7)	28 (66.7)	<0.001
Do (did) you feel well prepared for labour and birth?	39 (100.0)	36 (90.0)	0.128	41 (97.6)	34 (85.0)	0.099
**Social support**						
Do you have someone						
to socialise with	38 (97.4)	36 (100.0)	1.000	42 (100.0)	41 (100.0)	NA
to talk with about your problems	38 (97.4)	37 (100.0)	1.000	42 (100.0)	41 (100.0)	NA
to help you if you were sick and needed to be in bed	38 (97.4)	37 (100.0)	1.000	42 (100.0)	41 (100.0)	NA
who can lend you 500 SEK if you have a sudden need?	38 (97.4)	35 (94.6)	0.963	42 (100.0)	41 (100.0)	NA
who can take care of your children for a while if needed?	37 (97.4)	29 (96.7)	1.000	42 (100.0)	38 (97.4)	0.970
who can help you with a temporary place to live next year if you should need it	31 (79.5)	28 (75.7)	0.902	40 (95.2)	18 (43.9)	<0.001
Have you made new friends through the antenatal care clinic during your pregnancy?[2]				41 (97.6)	10 (25.0)	<0.001
Have you felt lonely and isolated since you had the baby?[2]				1 (2.4)	2 (4.9)	0.983

[1]Only in questionnaire 1

[2]Only in questionnaire 2

For peer review only

For peer review only - http://bmjopen.bmj.com/site/about/guidelines.xhtml

Knowledge of danger signs

Knowledge of danger signs and who to contact if experiencing severe symptoms, was assessed in late pregnancy (not presented in table). A significantly higher proportion of women in gANC responded that they would contact the delivery ward in case of vaginal bleeding (gANC 54.0%; sANC 28.8%; p=0.004) or leakage of amniotic fluid (gANC 55.6%; sANC 26.0%; p=0.003). For severe headache and changes in vision, 46.0% of women in gANC said they would contact the emergency room compared to 23.3% of women in standard care (p=0.037). Of women in gANC, 30.2% responded that they would contact the delivery ward if experiencing reduced fetal movements, compared to 17.8% of women in sANC (p=0.526).

Obstetric outcomes

Obstetric outcomes in relation to care model are described in Table 4. Compared with women in standard care, women in gANC made more visits to the antenatal clinic and to specialist care, however, the other health parameters were similar between the groups. Labour and birth outcomes were also similar between the groups, such as induction of labour, pain relief, perineal injury, blood loss and length of stay.

For peer review only

For peer review only - http://bmjopen.bmj.com/site/about/guidelines.xhtml

Table 4. Obstetric outcomes in relation to care model

	Group antenatal care n=63		Standard care n=73		
	n (%)	median, IQR	n (%)	median, IQR	p-value
**Antenatal care**					
Number of ANC visits[1,2] (median, IQR)		9.5 (8.00; 11.00)		9.0 (7.25; 10.00)	0.022
≤6	3 (5.6)		2 (3.0)		0.018
7-11	40 (74.1)		61 (92.4)		
>11	11 (20.4)		3 (4.5)		
Number of visits to specialist care (median, IQR)		2.00 (1.00; 5.00)		1.00 (0.00; 3.00)	0.014
Number of women referred to a specialist (obstetrician)	49 (90.7)		51 (77.3)		0.085
Screened for experience of violence[3]	37 (88.1)		37 (84.1)		0.822
Attendance at parent education[4]	9 (27.3)		3 (8.3)		0.056
**Health parameters**					
Haemoglobin (lowest value)		111 (106; 118)		108 (104; 114)	0.181
Haemoglobin (last value prior to birth)		122 (118; 128)		120 (115; 126)	0.218
S-Ferritin (lowest value)		24 (16; 54)		23 (13; 42)	0.539
Weight gain during pregnancy (kg)		8.0 (4.3; 12.0)		7.0 (4.0; 12.0)	0.589
Gestational diabetes mellitus	7 (11.1)		7 (9.6)		0.771
**Labour and birth**					
Induction of labour	8 (17.8)		10 (16.4)		1.000
Oxytocin for dystocia	16 (42.1)		23 (37.7)		0.823
Pain relief					
Epidural (EDA)	9 (14.5)		5 (7.4)		0.259
Gas (Nitrous oxide)	33 (53.2)		35 (50.7)		0.912
Other (TENS, sterile water injection, bath, PCB, PDB)	1 (1.6)		2 (2.9)		1.000
Mode of birth					
Vaginal	42 (77.8)		52 (78.8)		0.064
Assisted vaginal delivery	0 (0.0)		6 (9.1)		
Elective caesarean	8 (14.8)		4 (6.1)		
Emergency caesarean	4 (7.4)		4 (6.1)		
Perineal injury (degree III-IV)	0 (0.0)		4 (5.8)		0.121
Blood loss (within 2 hours) (ml)		480 (300; 650)		400 (300; 538)	0.345
Breastfeeding on delivery ward	50 (98.0)		61 (96.8)		1.000
Length of stay (in hospital after birth)		1.00 (0.0; 2.0)		1.00 (0.0; 2.3)	0.590
Discharged the same day as giving birth	23 (43.4)		30 (46.9)		0.850
**Infant outcomes**					
Live births[5]	62		73		
Stillborn	1 (1.6)		1 (1.4)		1.000
Gestational age (weeks)		40.0 (39.0; 40.0)		40.0 (39.0; 40.3)	0.989
Birthweight (grams)		3580 (3180; 3778)		3490 (3166; 3776)	0.839
Small for gestational age	7 (11.1)		0 (0.0)		0.011
Large for gestational age	3 (4.7)		2 (2.7)		0.532
Apgar score <7 at 5 minutes	0 (0.0)		1 (1.7)		1.000
Umbilical cord pH (arterial)(median, IQR)		7.24 (7.20; 7.31)		7.26 (7.23; 7.31)	0.224
Umbilical cord pH (vein)(median, IQR)		7.35 (7.29; 7.37)		7.35 (7.30; 7.37)	0.795
Neonatal intensive care	1 (1.6)		5 (7.2)		0.212
**Postnatal check-up**					
Postnatal visit	51 (81.0)		47 (64.4)		0.032
Fully breastfeeding at 4 weeks after birth	25 (39.7)		24 (32.9)		0.410
Body Mass Index (kg/m2) at postnatal check-up visit		28.34 (25.56;31.64)		29.63 (26.44; 33.12)	0.379

[1]Not adjusted for gestational age.

[2]The recommended number of prenatal visits in Sweden is 9 for a normal 40-week pregnancy

For peer review only

For peer review only - http://bmjopen.bmj.com/site/about/guidelines.xhtml

[3]Screening for violence is recommended in the Swedish National Guidelines for ANC

[4]Other than group antenatal care

[5]One twin birth in standard care

Discussion

The aim of this study was to evaluate a model of gANC for Somali-speaking women in Sweden. No differences regarding overall ratings of antenatal care were detected, however, the reduction in mean EPDS-score was greater in the intervention group when adjusting for differences at baseline. In late pregnancy, women in gANC were happier with received pregnancy and birth information compared to women in standard care.

Participants in gANC rated their antenatal care experience at least on par with women who received standard antenatal care. The decrease in EPDS mean scores was larger in women in gANC than in sANC, adjusted for differences at baseline. The gANC group had relatively high EPDS median scores (10), when entering the intervention, and higher than the control group (6), indicating a probable selection bias. It is possible that women who were feeling vulnerable and wanting more support and information from ANC were more interested in participation in gANC. Women who participated in gANC had comparatively low levels of education, no previous births in Sweden, were less involved in working life and perhaps less knowledgeable about Swedish health care. Whether or not the larger reduction in mean EPDS scores among women in gANC can be attributed to the intervention or to other aspects of care given the study design, the reduced sample-size and the above discussion, is uncertain. In a small Norwegian study on postpartum depression among Somali women, the mean EPDS score was 2.97 (SD 3.31), which is very low, and might reflect a different understanding of the EPDS questions (69) or a selection of women other than in the present study. In our case, the EPDS questions were asked by the research assistant rather than women completing the EPDS themselves which may have impacted the responses, but it did allow for clarification of items, if they were not clear. Information on history of mental illness or medication was not collected. A still unpublished Swedish study suggests that the Somali version of the EPDS has some major lexical problems. During think-aloud interviews, some Somali-speaking women left items unanswered when responding to the EPDS because they did not understand the meaning of them (oral communication Schytt el al), which may also explain some of the extensive missing rates in other surveys using the scale. Challenges with interpretation in research on mental ill health and cultural aspects of mental health also needs to be considered (70). The EPDS has been translated into Somali but is not yet validated (62), so in the absence

For peer review only - http://bmjopen.bmj.com/site/about/guidelines.xhtml

of validation, caution is required when interpreting EPDS for this group. Additional or other outcome measures than the EPDS might be appropriate in future trials to evaluate the effects of gANC on Somali women's emotional wellbeing.

The most interesting differences detected between the gANC and sANC groups were related to secondary outcomes. Women attending gANC were more likely to say that they had received sufficient information than women in sANC on most aspects of childbirth studied. Findings on the women's knowledge of danger signs were also promising. A higher proportion of women in gANC gained a better understanding of which health care facility to turn to for danger signs like vaginal bleeding and leakage of amniotic fluid. Information and education have been identified as essential in midwifery-led antenatal care, for adequate knowledge and for women to better understand the health care system, and women prefer health professionals who combine clinical knowledge and skills with interpersonal and cultural competence (71). In this study, as in others, Somali-born women had a high prevalence of risk factors during pregnancy, for example previous perinatal mortality and obesity. Our findings suggest some potential for gANC to improve information provision and knowledge acquisition among pregnant Somali-born women, especially women resident in Sweden <10 years.

Additionally, gANC may have played a role in strengthening social networks as intended. Nearly all women in gANC said they had someone who could help with a temporary place to live should they need it, compared to 25.0% of women in standard care two months postpartum. Additionally, nearly all women in gANC reported that they had made new friends through the ANC clinic during pregnancy, compared to only half of women in standard care.

The women in gANC had more appointments with the midwife compared to women in standard care, possibly because individual appointments were shorter in gANC. As a result, additional appointments were booked, rather than extending the time for each appointment when more time was needed. gANC is a complex intervention (72) and it is therefore not possible, nor appropriate, to determine the separate contributions of different components. The components have been studied together because of their hypothesised capacity jointly to make a difference to the provision of care. A case study from the US suggests that an underlying mechanism in which group prenatal care is effective is through increased quantity and quality of patient and practitioner time together and improved communication, fostering

For peer review only - http://bmjopen.bmj.com/site/about/guidelines.xhtml

greater opportunity for cross-cultural exposure and decreasing factors like the clinician´s implicit or explicit bias and racism (73). A process evaluation of the Hooyo-project has assessed the overall feasibility of developing, implementing and testing gANC for Somali-Swedish women, including the perspectives of the midwives, where the different components of this gANC model have been examined in more depth (74).

The inconsistent evidence about the impact of gANC on improved ratings of care and obstetric outcomes, may also be due to substantial differences between settings in the quality of standard antenatal care, as suggested in a Canadian study that found that group antenatal care was comparable to individual care on most outcomes measured (47). In the US however, there are greater disparities in the care received between subgroups, such as for women from minority groups or with low socioeconomic status who are uninsured (75). To achieve greater prenatal health equity for migrant women, there is a need to continue to investigate specific interventions in antenatal care focused on pregnant women and their families, on maternity care health professionals and on the system in which they work.

Strengths and limitations

This was the first controlled evaluation of gANC in Sweden attempting to address the particular needs of migrant women, with a participatory approach. This is of particular importance in groups of women who are often excluded in research, are hard to reach or when studying sensitive health issues (76). Moreover, this intervention and the evaluation were conducted in a real-world setting. Recruitment and retention of participants proved challenging. The recruitment pace was slower than anticipated and we had fewer eligible women than expected that registered in ANC and could not fill the groups in a satisfactory way. Recruitment had to be terminated before reaching the goal because of these factors in combination with resource limitations. We did not reach the goal of 70 women in each group, reducing the power to detect differences on overall rating of care between the groups. For the EPDS, however, we reached our recruitment goal of 63 women in each group. The two-stage recruitment process with midwives providing initial information and then subsequent recruitment of those showing interest by a bilingual research assistant may have made it harder to recruit women. Direct initial contact with a Somali-speaking research assistant might have made it easier for women to receive enough information in their own language to agree to participate. While the research assistants were pivotal in recruitment, retention and data collection in this study, women's responses were not given anonymously, which may

For peer review only

have affected participation and responses. Additionally, participation in research was likely to be unfamiliar for some of the women.

For this study, women were recruited from one site only, and the possible selection bias of participants recruited to the study further limits the generalisability of the results. We do not know if the results are representative for similar Somali-Swedish groups of women. The findings can however inform future randomised controlled trials and be useful for future power calculations.

Conclusions

This study suggests potential for gANC to improve information provision and knowledge acquisition among pregnant Somali-born women with residence in Sweden <10 years. The study found few significant differences between the groups on women's overall ratings of care and emotional wellbeing measured with EPDS. The study demonstrated feasibility of assessing the included outcomes with Somali-born women. Moreover, the study suggests that other outcomes measures are important when evaluating language-supported gANC, and that a validation of the Somali version of the EPDS is needed. Finally, an adequately powered randomised trial is needed for the effectiveness of gANC to be robustly assessed.

Acknowledgements

We are grateful to all the women and midwives who participated and shared their experiences at different stages of this project, and to the Reference Group. We wish to commemorate midwife Ros-Mari Casselbrant who passed away in October 2019. We wish to acknowledge the following contributors specifically: research assistants Fardosa Hassen Ahmed and Aisha Adan, interpreter Hamdi Sheek Hussein and David Grannas, statistician at Biostatistics Core Facility, Karolinska Institutet.

For peer review only

Contributors

All authors (MA, UB, ES, EA, RS, BE) were part of the research team that developed the intervention and the questionnaires. UB and MA monitored the day-to-day implementation of the intervention and data collection at the clinic. MA and ES were responsible for the analyses. All authors (MA, UB, ES, EA, RS, BE) have read, commented on and approved the final manuscript.

Competing interests

Conflict of interest: none to declare for any of the authors.

Funding

This work was supported by the Swedish Research Council (grant number 2015-02470), Forte (grant number 2016-00957) and the Doctoral School in Health Care Sciences, Karolinska Institutet (grant number N/A).

Data availability statement

Deidentified participant data are available upon reasonable request from the corresponding author, Malin Ahrne (malin.ahrne@ki.se).

Ethics approval

Ethical approval was obtained from Stockholm Ethical Review Board (2015/1703-31/1).

For peer review only

References

1. World Health Organization. WHO Recommendations on Antenatal Care for a Positive Pregnancy Experience. https://www.who.int/news/item/07-11-2016-new-guidelines-on-antenatal-care-for-a-positive-pregnancy-experience: World Health Organization; 2016.
2. The Sustainable Development Agenda https://www.un.org/sustainabledevelopment/development-agenda/: United Nations; 2015 [
3. Fair F, Raben L, Watson H, Vivilaki V, van den Muijsenbergh M, Soltani H. Migrant women's experiences of pregnancy, childbirth and maternity care in European countries: A systematic review. PLoS One. 2020;15(2):e0228378.
4. World Health Organization. Report on the health of refugees and migrants in the WHO European Region: no public health without refugee and migrant health. World Health Organization Regional Office for Europe; 2018.
5. International Organization for Migration (IOM). World Migration Report 2020. International Organisation of Migration IOM; 2019.
6. Villadsen SF, Mortensen LH, Andersen AM. Care during pregnancy and childbirth for migrant women: How do we advance? Development of intervention studies--the case of the MAMAACT intervention in Denmark. Best Pract Res Clin Obstet Gynaecol. 2016;32:100-12.
7. Rogers HJ, Hogan L, Coates D, Homer CSE, Henry A. Responding to the health needs of women from migrant and refugee backgrounds-Models of maternity and postpartum care in high-income countries: A systematic scoping review. Health Soc Care Community. 2020;28(5):1343-65.
8. Liu C, Ahlberg M, Hjern A, Stephansson O. Perinatal health of refugee and asylum-seeking women in Sweden 2014-17: a register-based cohort study. Eur J Public Health. 2019;29(6):1048-55.
9. Nilsen RM, Vik ES, Rasmussen SA, Small R, Moster D, Schytt E, et al. Preeclampsia by maternal reasons for immigration: a population-based study. BMC pregnancy and childbirth. 2018;18(1):423.
10. Gagnon AJ, Zimbeck M, Zeitlin J, Alexander S, Blondel B, Buitendijk S, et al. Migration to western industrialised countries and perinatal health: a systematic review. Soc Sci Med. 2009;69(6):934-46.
11. Bastola K, Koponen P, Gissler M, Kinnunen TI. Differences in caesarean delivery and neonatal outcomes among women of migrant origin in Finland: A population-based study. Paediatr Perinat Epidemiol. 2020;34(1):12-20.
12. Bastola K, Koponen P, Härkänen T, Luoto R, Gissler M, Kinnunen TI. Delivery and its complications among women of Somali, Kurdish, and Russian origin, and women in the general population in Finland. Birth. 2019;46(1):35-41.
13. Khanolkar AR, Wedrén S, Essén B, Sparén P, Koupil I. Preterm and postterm birth in immigrant- and Swedish-born parents: a population register-based study. Eur J Epidemiol. 2015;30(5):435-47.
14. Bakken KS, Skjeldal OH, Stray-Pedersen B. Higher risk for adverse obstetric outcomes among immigrants of African and Asian descent: a comparison study at a low-risk maternity hospital in Norway. Birth. 2015;42(2):132-40.
15. Almeida LM, Caldas J, Ayres-de-Campos D, Salcedo-Barrientos D, Dias S. Maternal healthcare in migrants: a systematic review. Matern Child Health J. 2013;17(8):1346-54.
16. Belihu FB, Davey MA, Small R. Perinatal health outcomes of East African immigrant populations in Victoria, Australia: a population based study. BMC pregnancy and childbirth. 2016;16:86.
17. Malin M, Gissler M. Maternal care and birth outcomes among ethnic minority women in Finland. BMC Public Health. 2009;9:84.
18. Vik ES, Aasheim V, Schytt E, Small R, Moster D, Nilsen RM. Stillbirth in relation to maternal country of birth and other migration related factors: a population-based study in Norway. BMC pregnancy and childbirth. 2019;19(1):5.

For peer review only - http://bmjopen.bmj.com/site/about/guidelines.xhtml

19. Råssjö EB, Byrskog U, Samir R, Klingberg-Allvin M. Somali women's use of maternity health services and the outcome of their pregnancies: a descriptive study comparing Somali immigrants with native-born Swedish women. Sex Reprod Healthc. 2013;4(3):99-106.
20. Akselsson A, Lindgren H, Georgsson S, Pettersson K, Skokic V, Rådestad I. Pregnancy outcomes among women born in Somalia and Sweden giving birth in the Stockholm area - a population-based study. Global health action. 2020;13(1):1794107.
21. Essén B, Hanson BS, Ostergren PO, Lindquist PG, Gudmundsson S. Increased perinatal mortality among sub-Saharan immigrants in a city-population in Sweden. Acta Obstet Gynecol Scand. 2000;79(9):737-43.
22. Small R, Gagnon A, Gissler M, Zeitlin J, Bennis M, Glazier R, et al. Somali women and their pregnancy outcomes postmigration: data from six receiving countries. Bjog. 2008;115(13):1630-40.
23. Johnson EB, Reed SD, Hitti J, Batra M. Increased risk of adverse pregnancy outcome among Somali immigrants in Washington state. Am J Obstet Gynecol. 2005;193(2):475-82.
24. Oliver EA, Klebanoff M, Yossef-Salameh L, Oza-Frank R, Moosavinasab S, Reagan P, et al. Preterm Birth and Gestational Length in Four Race-Nativity Groups, Including Somali Americans. Obstet Gynecol. 2018;131(2):281-9.
25. Ahrne M, Schytt E, Andersson E, Small R, Adan A, Essén B, et al. Antenatal care for Somali-born women in Sweden: Perspectives from mothers, fathers and midwives. Midwifery. 2019;74:107-15.
26. Schmidt NC, Fargnoli V, Epiney M, Irion O. Barriers to reproductive health care for migrant women in Geneva: a qualitative study. Reprod Health. 2018;15(1):43.
27. Bains S, Skråning S, Sundby J, Vangen S, Sørbye IK, Lindskog BV. Challenges and barriers to optimal maternity care for recently migrated women - a mixed-method study in Norway. BMC pregnancy and childbirth. 2021;21(1):686.
28. Essén B, Johnsdotter S, Hovelius B, Gudmundsson S, Sjöberg NO, Friedman J, et al. Qualitative study of pregnancy and childbirth experiences in Somalian women resident in Sweden. Bjog. 2000;107(12):1507-12.
29. Small R, Roth C, Raval M, Shafiei T, Korfker D, Heaman M, et al. Immigrant and non-immigrant women's experiences of maternity care: a systematic and comparative review of studies in five countries. BMC pregnancy and childbirth. 2014;14:152.
30. Higginbottom GM, Morgan M, Alexandre M, Chiu Y, Forgeron J, Kocay D, et al. Immigrant women's experiences of maternity-care services in Canada: a systematic review using a narrative synthesis. Syst Rev. 2015;4:13.
31. Heslehurst N, Brown H, Pemu A, Coleman H, Rankin J. Perinatal health outcomes and care among asylum seekers and refugees: a systematic review of systematic reviews. BMC Med. 2018;16(1):89.
32. Heaman M, Bayrampour H, Kingston D, Blondel B, Gissler M, Roth C, et al. Migrant women's utilization of prenatal care: a systematic review. Matern Child Health J. 2013;17(5):816-36.
33. Essén B, Bödker B, Sjöberg NO, Langhoff-Roos J, Greisen G, Gudmundsson S, et al. Are some perinatal deaths in immigrant groups linked to suboptimal perinatal care services? Bjog. 2002;109(6):677-82.
34. Beine K, Fullerton J, Palinkas L, Anders B. Conceptions of prenatal care among Somali women in San Diego. J Nurse Midwifery. 1995;40(4):376-81.
35. Davies MM, Bath PA. The maternity information concerns of Somali women in the United Kingdom. J Adv Nurs. 2001;36(2):237-45.
36. Wiklund H, Aden AS, Högberg U, Wikman M, Dahlgren L. Somalis giving birth in Sweden: a challenge to culture and gender specific values and behaviours. Midwifery. 2000;16(2):105-15.
37. Herrel N, Olevitch L, DuBois DK, Terry P, Thorp D, Kind E, et al. Somali refugee women speak out about their needs for care during pregnancy and delivery. J Midwifery Womens Health. 2004;49(4):345-9.
38. Esscher A, Binder-Finnema P, Bodker B, Hogberg U, Mulic-Lutvica A, Essen B. Suboptimal care and maternal mortality among foreign-born women in Sweden: maternal death audit with application of the 'migration three delays' model. BMC pregnancy and childbirth. 2014;14:141.

For peer review only - http://bmjopen.bmj.com/site/about/guidelines.xhtml

39. Ny P, Dykes AK, Molin J, Dejin-Karlsson E. Utilisation of antenatal care by country of birth in a multi-ethnic population: a four-year community-based study in Malmö, Sweden. Acta Obstet Gynecol Scand. 2007;86(7):805-13.
40. Fabian H, Rådestad I, Rodriguez A, Waldenström U. Women with non-Swedish speaking background and their children: a longitudinal study of uptake of care and maternal and child health. Acta Paediatr. 2008;97(12):1721-8.
41. Fabian HM, Rådestad IJ, Waldenström U. Characteristics of Swedish women who do not attend childbirth and parenthood education classes during pregnancy. Midwifery. 2004;20(3):226-35.
42. Ludwig A, Miani C, Breckenkamp J, Sauzet O, Borde T, Doyle IM, et al. Are Social Status and Migration Background Associated with Utilization of Non-medical Antenatal Care? Analyses from Two German Studies. Matern Child Health J. 2020;24(7):943-52.
43. Catling CJ, Medley N, Foureur M, Ryan C, Leap N, Teate A, et al. Group versus conventional antenatal care for women. Cochrane Database Syst Rev. 2015;2015(2):Cd007622.
44. Carter EB, Temming LA, Akin J, Fowler S, Macones GA, Colditz GA, et al. Group Prenatal Care Compared With Traditional Prenatal Care: A Systematic Review and Meta-analysis. Obstet Gynecol. 2016;128(3):551-61.
45. Hunter L, Da Motta G, McCourt C, Wiseman O, Rayment J, Haora P, et al. `It makes sense and it works': Maternity care providers' perspectives on the feasibility of a group antenatal care model (Pregnancy Circles). Midwifery. 2018;66:56-63.
46. Cunningham SD, Lewis JB, Thomas JL, Grilo SA, Ickovics JR. Expect With Me: development and evaluation design for an innovative model of group prenatal care to improve perinatal outcomes. BMC pregnancy and childbirth. 2017;17(1):147.
47. Hodgson ZG, Saxell L, Christians JK. An evaluation of Interprofessional group antenatal care: a prospective comparative study. BMC pregnancy and childbirth. 2017;17(1):297.
48. Cunningham SD, Grilo S, Lewis JB, Novick G, Rising SS, Tobin JN, et al. Group Prenatal Care Attendance: Determinants and Relationship with Care Satisfaction. Matern Child Health J. 2017;21(4):770-6.
49. Liu R, Chao MT, Jostad-Laswell A, Duncan LG. Does CenteringPregnancy Group Prenatal Care Affect the Birth Experience of Underserved Women? A Mixed Methods Analysis. J Immigr Minor Health. 2017;19(2):415-22.
50. Byerley BM, Haas DM. A systematic overview of the literature regarding group prenatal care for high-risk pregnant women. BMC pregnancy and childbirth. 2017;17(1):329.
51. Felder JN, Epel E, Lewis JB, Cunningham SD, Tobin JN, Rising SS, et al. Depressive symptoms and gestational length among pregnant adolescents: Cluster randomized control trial of CenteringPregnancy® plus group prenatal care. J Consult Clin Psychol. 2017;85(6):574-84.
52. Hollowell J, Oakley L, Kurinczuk JJ, Brocklehurst P, Gray R. The effectiveness of antenatal care programmes to reduce infant mortality and preterm birth in socially disadvantaged and vulnerable women in high-income countries: a systematic review. Bmc Pregnancy and Childbirth. 2011;11.
53. Andersson E, Christensson K, Hildingsson I. Mothers' satisfaction with group antenatal care versus individual antenatal care--a clinical trial. Sex Reprod Healthc. 2013;4(3):113-20.
54. Riggs E, Muyeen S, Brown S, Dawson W, Petschel P, Tardiff W, et al. Cultural safety and belonging for refugee background women attending group pregnancy care: An Australian qualitative study. Birth. 2017;44(2):145-52.
55. Madeira AD, Rangen CM, Avery MD. Design and Implementation of a Group Prenatal Care Model for Somali Women at a Low-Resource Health Clinic. Nurs Womens Health. 2019;23(3):224-33.
56. Statistics Sweden https://www.scb.se/hitta-statistik/sverige-i-siffror/manniskorna-i-sverige/utrikes-fodda/ 2021 [
57. Byrskog U, Ahrne M, Small R, Andersson E, Essen B, Adan A, et al. Rationale, development and feasibility of group antenatal care for immigrant women in Sweden: a study protocol for the Hooyo Project. BMJ Open. 2019;9(7):e030314.
58. Swedish Society of Obstetrics and Gynecology (SFOG). Mödrahälsovård, Sexuell och Reproduktiv Hälsa (Prenatal Care, Sexual and Repriductive Health) (Published in 2008, updated as a

For peer review only - http://bmjopen.bmj.com/site/about/guidelines.xhtml

web-version in 2016). https://www.sfog.se/natupplaga/ARG76web4a328b70-0d76-474e-840e-31f70a89eae9.pdf; 2016.
59. Gagnon AJ, DeBruyn R, Essen B, Gissler M, Heaman M, Jeambey Z, et al. Development of the Migrant Friendly Maternity Care Questionnaire (MFMCQ) for migrants to Western societies: an international Delphi consensus process. BMC Pregnancy Childbirth. 2014;14:200.
60. Cox J. Thirty years with the Edinburgh Postnatal Depression Scale: voices from the past and recommendations for the future. Br J Psychiatry. 2019;214(3):127-9.
61. Rubertsson C, Börjesson K, Berglund A, Josefsson A, Sydsjö G. The Swedish validation of Edinburgh Postnatal Depression Scale (EPDS) during pregnancy. Nord J Psychiatry. 2011;65(6):414-8.
62. Cox J HJ, Henshaw C, et al. . The Edinburgh Postnatal Depression Scale (EPDS) Manual. 2 edn. : The Royal College of psychiatrists; 2012.
63. Morkved S, Bo K. Prevalence of urinary incontinence during pregnancy and postpartum. Int Urogynecol J Pelvic Floor Dysfunct. 1999;10(6):394-8.
64. PRAMS Questionnaires (Pregnancy Risk Assessment Monitoring System) Phase 8 Standard Questions February 2017 https://www.cdc.gov/prams/questionnaire.htm#current: Centers for Disease Control and Prevention (CDC); 2017 [
65. Green JM, Kafetsios K, Statham HE, Snowdon CM. Factor structure, validity and reliability of the Cambridge Worry Scale in a pregnant population. J Health Psychol. 2003;8(6):753-64.
66. Hildingsson I, Rådestad I. Swedish women's satisfaction with medical and emotional aspects of antenatal care. J Adv Nurs. 2005;52(3):239-49.
67. Rubertsson C, Wickberg B, Gustavsson P, Rådestad I. Depressive symptoms in early pregnancy, two months and one year postpartum-prevalence and psychosocial risk factors in a national Swedish sample. Arch Womens Ment Health. 2005;8(2):97-104.
68. Gupta SK. Intention-to-treat concept: A review. Perspect Clin Res. 2011;2(3):109-12.
69. Løvlie AL, Madar AA. Postpartum Depression Among Somali Women in Norway. J Immigr Minor Health. 2017;19(3):638-44.
70. Ingvarsdotter K, Johnsdotter S, Östman M. Lost in interpretation: the use of interpreters in research on mental ill health. Int J Soc Psychiatry. 2012;58(1):34-40.
71. Renfrew MJ, McFadden A, Bastos MH, Campbell J, Channon AA, Cheung NF, et al. Midwifery and quality care: findings from a new evidence-informed framework for maternal and newborn care. Lancet. 2014;384(9948):1129-45.
72. Moore GF, Audrey S, Barker M, Bond L, Bonell C, Hardeman W, et al. Process evaluation of complex interventions: Medical Research Council guidance. BMJ. 2015;350:h1258.
73. Carter EB, Mazzoni SE. A paradigm shift to address racial inequities in perinatal healthcare. Am J Obstet Gynecol. 2021;224(4):359-61.
74. Ahrne M, Byrskog U, Essén B, Andersson E, Small R, Schytt E. Group antenatal care (gANC) for Somali-speaking women in Sweden - a process evaluation. BMC pregnancy and childbirth. 2022;22(1):721.
75. Park JH, Vincent D, Hastings-Tolsma M. Disparity in prenatal care among women of colour in the USA. Midwifery. 2007;23(1):28-37.
76. Ismail MM, Gerrish K, Naisby A, Salway S, Chowbey P. Engaging minorities in researching sensitive health topics by using a participatory approach. Nurse Res. 2014;22(2):44-8.

For peer review only - http://bmjopen.bmj.com/site/about/guidelines.xhtml

Figure legends

Figure 1. Women´s overall ratings of ANC care

The proportion of women who were "always", "mostly", "sometimes", "rarely" and "never" happy with the care they received in the two different care models, assessed in late pregnancy and two months postpartum.

Figure 2. Median scores on the Edinburgh Postnatal Depression Scale (EPDS)

At baseline (Q1), late pregnancy (Q2) and 2 months postpartum (Q3), analysed by ANCOVA test of differences. The boxplot also shows minimum and maximum score, and the 1st and 3rd quartile and the mean differences with 95% CI at each time point.

For peer review only

For peer review only - http://bmjopen.bmj.com/site/about/guidelines.xhtml

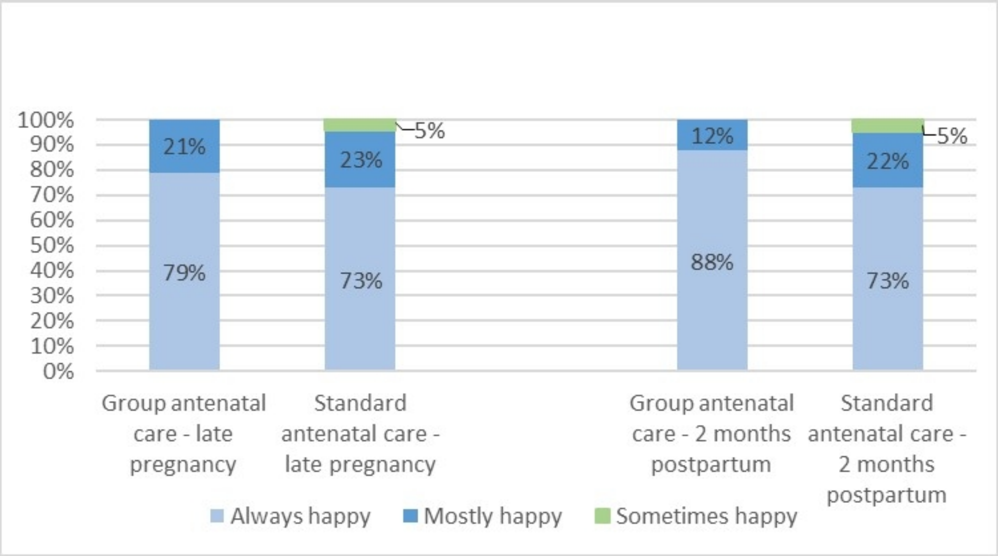

Figure 1. Women´s overall ratings of ANC care – the proportion of women who were "always", "mostly", "sometimes", "rarely" and "never" happy with the care they received in the two different care models, assessed in late pregnancy and two months postpartum.

127x70mm (150 x 150 DPI)

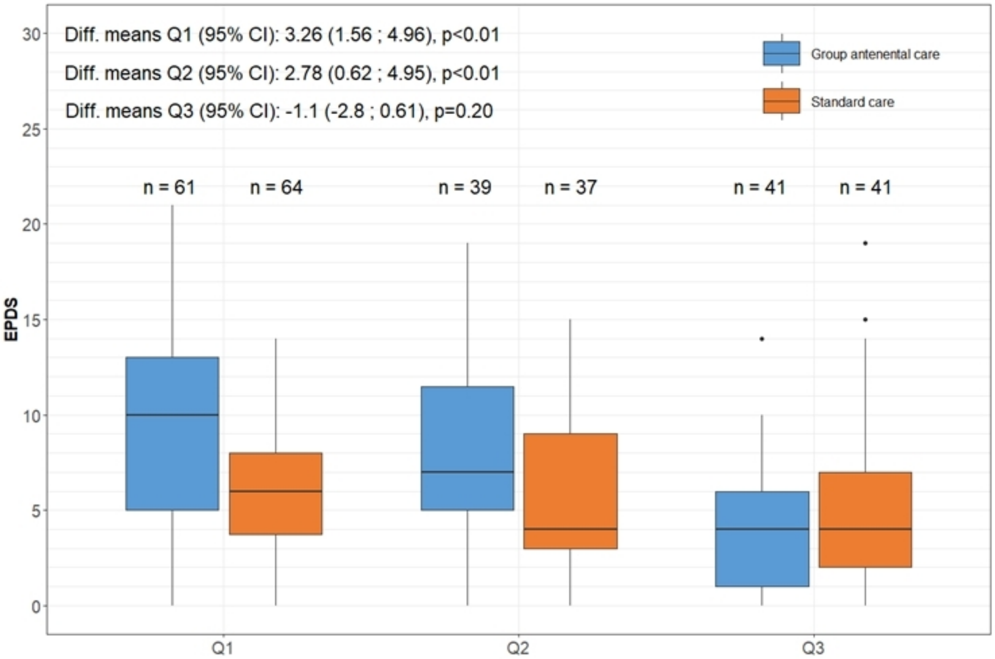

Figure 2. Median scores on the Edinburgh Postnatal Depression Scale (EPDS); at baseline (Q1), late pregnancy (Q2) and 2 months postpartum (Q3) analysed by ANCOVA test of differences. The boxplot also shows minimum and maximum score, and the 1st and 3rd quartile and the mean differences with 95% CI at each time point.

125x88mm (150 x 150 DPI)

CONSORT 2010 checklist of information to include when reporting a randomised trial*

Section/Topic	Item No	Checklist item	Reported on page No
**Title and abstract**			
	1a	Identification as a randomised trial in the title	1
	1b	Structured summary of trial design, methods, results, and conclusions (for specific guidance see CONSORT for abstracts)	3
**Introduction**			
Background and objectives	2a	Scientific background and explanation of rationale	5
	2b	Specific objectives or hypotheses	6
**Methods**			
Trial design	3a	Description of trial design (such as parallel, factorial) including allocation ratio	7
	3b	Important changes to methods after trial commencement (such as eligibility criteria), with reasons	-
Participants	4a	Eligibility criteria for participants	7
	4b	Settings and locations where the data were collected	7
Interventions	5	The interventions for each group with sufficient details to allow replication, including how and when they were actually administered	8+9
Outcomes	6a	Completely defined pre-specified primary and secondary outcome measures, including how and when they were assessed	9+10
	6b	Any changes to trial outcomes after the trial commenced, with reasons	-
Sample size	7a	How sample size was determined	11
	7b	When applicable, explanation of any interim analyses and stopping guidelines	-
Randomisation:			-
Sequence generation	8a	Method used to generate the random allocation sequence	
	8b	Type of randomisation; details of any restriction (such as blocking and block size)	-
Allocation concealment mechanism	9	Mechanism used to implement the random allocation sequence (such as sequentially numbered containers), describing any steps taken to conceal the sequence until interventions were assigned	-
Implementation	10	Who generated the random allocation sequence, who enrolled participants, and who assigned participants to interventions	-
Blinding	11a	If done, who was blinded after assignment to interventions (for example, participants, care providers, those	-

For peer review only - http://bmjopen.bmj.com/site/about/guidelines.xhtml

		assessing outcomes) and how	
	11b	If relevant, description of the similarity of interventions	-
Statistical methods	12a	Statistical methods used to compare groups for primary and secondary outcomes	11
	12b	Methods for additional analyses, such as subgroup analyses and adjusted analyses	-
**Results**			
Participant flow (a diagram is strongly recommended)	13a	For each group, the numbers of participants who were randomly assigned, received intended treatment, and were analysed for the primary outcome	12
	13b	For each group, losses and exclusions after randomisation, together with reasons	12
Recruitment	14a	Dates defining the periods of recruitment and follow-up	12
	14b	Why the trial ended or was stopped	12
Baseline data	15	A table showing baseline demographic and clinical characteristics for each group	12-13
Numbers analysed	16	For each group, number of participants (denominator) included in each analysis and whether the analysis was by original assigned groups	14+15
Outcomes and estimation	17a	For each primary and secondary outcome, results for each group, and the estimated effect size and its precision (such as 95% confidence interval)	14+15
	17b	For binary outcomes, presentation of both absolute and relative effect sizes is recommended	-
Ancillary analyses	18	Results of any other analyses performed, including subgroup analyses and adjusted analyses, distinguishing pre-specified from exploratory	-
Harms	19	All important harms or unintended effects in each group (for specific guidance see CONSORT for harms)	-
**Discussion**			
Limitations	20	Trial limitations, addressing sources of potential bias, imprecision, and, if relevant, multiplicity of analyses	23
Generalisability	21	Generalisability (external validity, applicability) of the trial findings	24
Interpretation	22	Interpretation consistent with results, balancing benefits and harms, and considering other relevant evidence	-
**Other information**			
Registration	23	Registration number and name of trial registry	7
Protocol	24	Where the full trial protocol can be accessed, if available	7
Funding	25	Sources of funding and other support (such as supply of drugs), role of funders	25

*We strongly recommend reading this statement in conjunction with the CONSORT 2010 Explanation and Elaboration for important clarifications on all the items. If relevant, we also recommend reading CONSORT extensions for cluster randomised trials, non-inferiority and equivalence trials, non-pharmacological treatments, herbal interventions, and pragmatic trials. Additional extensions are forthcoming: for those and for up to date references relevant to this checklist, see www.consort-statement.org.

For peer review only - http://bmjopen.bmj.com/site/about/guidelines.xhtml
